# Processing of double-R-loops in (CAG)·(CTG) and *C9orf72* (GGGGCC)·(GGCCCC) repeats causes instability

**DOI:** 10.1093/nar/gku658

**Published:** 2014-08-21

**Authors:** Kaalak Reddy, Monika H.M. Schmidt, Jaimie M. Geist, Neha P. Thakkar, Gagan B. Panigrahi, Yuh-Hwa Wang, Christopher E. Pearson

**Affiliations:** 1 Department of Genetics, The Hospital for Sick Children, Peter Gilgan Centre for Research & Learning, 686 Bay Street, Toronto, Ontario M5G 0A4, Canada; 2 Program of Molecular Genetics, University of Toronto, Toronto, Ontario M5G 0A4, Canada; 3 Department of Biology, Laurentian University, Sudbury, Ontario P3E 2C6, Canada; 4 Department of Biochemistry & Molecular Genetics, University of Virginia School of Medicine, Charlottesville, VA 22908, USA

## Abstract

R-loops, transcriptionally-induced RNA:DNA hybrids, occurring at repeat tracts (CTG)*_n_*, (CAG)_*n*_, (CGG)_*n*_, (CCG)*_n_* and (GAA)*_n_*, are associated with diseases including myotonic dystrophy, Huntington's disease, fragile X and Friedreich's ataxia. Many of these repeats are bidirectionally transcribed, allowing for single- and double-R-loop configurations, where either or both DNA strands may be RNA-bound. R-loops can trigger repeat instability at (CTG)·(CAG) repeats, but the mechanism of this is unclear. We demonstrate R-loop-mediated instability through processing of R-loops by HeLa and human neuron-like cell extracts. Double-R-loops induced greater instability than single-R-loops. Pre-treatment with RNase H only partially suppressed instability, supporting a model in which R-loops directly generate instability by aberrant processing, or via slipped-DNA formation upon RNA removal and its subsequent aberrant processing. Slipped-DNAs were observed to form following removal of the RNA from R-loops. Since transcriptionally-induced R-loops can occur in the absence of DNA replication, R-loop processing may be a source of repeat instability in the brain. Double-R-loop formation and processing to instability was extended to the expanded *C9orf72* (GGGGCC)·(GGCCCC) repeats, known to cause amyotrophic lateral sclerosis and frontotemporal dementia, providing the first suggestion through which these repeats may become unstable. These findings provide a mechanistic basis for R-loop-mediated instability at disease-associated repeats.

## INTRODUCTION

R-loops are thermodynamically stable, RNA:DNA structures that may form during transcription. As nascent RNA is produced within the transcription bubble, short stretches of RNA:DNA hybrids are transiently formed with the parental DNA strand and lost with subsequent ejection from the transcribing RNA polymerase (1). At certain GC-rich DNA sequences, stable R-loops are formed and persist following passage of the RNA polymerase along the transcribed DNA template due to the high stability of rG·dC base pairs between RNA and DNA, preferentially occurring when G clusters are present in the transcript (2,3). Biological roles for R-loops have been described in DNA replication initiation at mitochondrial and prokaryotic origins of replication, in class switch recombination at immunoglobulin genes, and in DNA methylation regulation at CpG island sequences (4–7). In these cases, the formation of R-loops is a necessary event regulating essential downstream activities. However, R-loops can also act as mutagenic intermediates and can cause either gene-specific or genome-wide instability. For example, mutations in the THO/TREX complex, which is required for proper coupling of transcription and mRNA export, cause wide-scale co-transcriptional R-loop formation triggering aberrant recombination leading to genome-wide instability (8–10). In contrast, gene-specific R-loop-mediated mutagenesis may have specific sequence requirements, as described below.

Certain sequences prone to R-loop formation, such as at expanded (CTG)·(CAG) repeat tracts, can trigger instability in a gene-specific manner (11,12). Gene-specific instability of trinucleotide repeat sequences is the causative mutation for various neurological, neuromuscular and neurodegenerative diseases (13). At least 14 diseases are caused by gene-specific expansions of (CAG)·(CTG) repeats including Huntington's disease, myotonic dystrophy type 1 and a series of spinocerebellar ataxias. Others include fragile X mental retardation involving (CGG)·(CCG) repeats and Friedreich's ataxia involving a (GAA)·(TTC) repeat (14). Recently, the leading genetic cause of amyotrophic lateral sclerosis (ALS) and frontotemporal dementia (FTD) has been linked to an expanded (GGGGCC)·(GGCCCC) repeat in the *C9orf72* gene which also shows length variation between tissues of a given individual (15). In many instances, these disease-associated repeats, including the *C9orf72* tract, are transcribed in either one or both directions (16,17). Evidence suggests that transcription across the repeat tract drives repeat instability (18–21). Notably, simultaneous bidirectional transcription can exacerbate genetic instability of a (CAG)·(CTG) repeat tract in human cells (21–23). Transcription across CAG, CTG, CGG, CCG and GAA repeats can lead to the formation of single-R-loops, where the RNA transcript is retained in a stable hybrid with the DNA and the non-template DNA strand is rendered single-stranded (3,11,24). We previously demonstrated the formation of novel double-R-loop structures in which both strands of a convergent bidirectionally transcribed (CAG)·(CTG) repeat tract or a (CGG)·(CCG) repeat tract contained RNA:DNA hybrids on both strands, termed double-R-loops (3). Double-R-loops may be associated with the increased instability observed during bidirectional transcription (22,23).

Transcriptionally-induced R-loops on expanded CAG repeat tracts was coincident with increased levels of trinucleotide repeat contractions. Knockdown of RNase H1 and H2 enzymes led to even greater contraction frequency in the repeat tract, further supporting the role of R-loops in instability (11). Interestingly, we found that upon removal of the RNA with RNase H, from both single- and double-R-loops, a portion of the DNA template remained single-stranded (3). Single-stranded regions of the repeat tract may assume slipped-DNA (S-DNA) structures formed from misalignment during re-annealing of the separated DNA strands, which may trigger instability, as previously hypothesized (11,25).

Although previous findings established a connection between transcription, R-loops and instability, there are important unanswered questions regarding the mechanistic basis of R-loop-mediated instability. For example, can R-loops be directly processed post-transcriptionally in the absence of DNA replication? This is an important question because instability occurs in various non-proliferative cells such as neurons (14). Also, since many (CAG)·(CTG) repeat tracts are transcribed in both directions, does the configuration of the R-loop (rCAG-containing R-loop, rCUG-containing R-loop or rCAG+rCUG-containing double-R-loops) influence the instability of the repeat tract? Finally, might R-loop-mediated instability involve an S-DNA intermediate produced through R-loop degradation by RNase H elimination of the RNA portion? Addressing these questions regarding R-loop processing has proven difficult because there are currently no suitable assays to directly elucidate the mechanism of R-loop processing upon (CAG)·(CTG) repeat instability.

In this study, we establish an *in vitro* R-loop processing assay using human cell extracts and demonstrate that R-loops and double-R-loops formed at an expanded (CAG)_79_·(CTG)_79_ repeat tract can lead to repeat instability post-transcriptionally and in the absence of DNA replication. Similarly, we show that a (GGGGCC)*_n_*·(GGCCCC)*_n_* repeat tract from the *C9orf72* gene with expansion lengths ranging from 4 to 94 repeats was able to form R- and double-R-loops. We show that double-R-loops arising from simultaneous convergent bidirectional transcription, cause the greatest instability relative to either single-R-loop alone. Finally, R-loop degradation by RNase H only partially suppresses the observed instability likely due to the formation of S-DNA structures within the repeat tract. In support of this model, we also reveal that removal of the RNA entity from R-loops by RNase H can induce S-DNA formation. These findings shed light on the mechanistic basis for R-loop-mediated instability at disease-associated repeat tracts.

## MATERIALS AND METHODS

### *In vitro* transcription and RNase treatments to generate R-loop templates

Plasmids bearing an expanded (CAG)_79_·(CTG)_79_ repeat tract with convergent T3 and T7 RNA polymerase promoters are described in detail previously (26). Large-scale plasmid preparations were prepared from dam+ *Escherichia coli* cells as described previously (25). Briefly, cells were harvested and lysed with lysozyme (Invitrogen) and a detergent solution of 1% Brij 58 (Sigma) and 0.4% deoxycholate (Sigma). Plasmids were treated with RNase A and T1 (Sigma), phenol-extracted and purified twice by cesium chloride/ethidium bromide centrifugation.

(GGGGCC)*_n_*·(GGCCCC)*_n_* clones, where *n* = 13, 21, 40 or 60 repeats (provided by Tao Zu and Laura Ranum as previously described (27), were prepared and propagated in DH5α cells and prepared as above. Requests for these clones must be directed to Dr Ranum.

Transcription reactions were performed as described (28,29). Briefly, 500 ng of template DNA in 1x transcription buffer (Roche) and 1x bovine serum albumin (NEB) were mixed in a final volume of 100 μl for 1 h with 20 U of the appropriate RNA polymerase: T7, T3 or T7+T3 (Roche). Total nucleic acid material was subsequently extracted with phenol/chloroform, then chloroform extraction followed by precipitation with 100% ethanol and 3 mM sodium acetate. Samples were resuspended in 10 μl 1x TE for subsequent RNase treatments. For incorporation of radioactive nucleotides, each transcription reaction was additionally carried out in the presence of 3.5 μCi of [α-^32^P]rCTP. Samples were run through sephadex G-50 columns (GE Healthcare) prior to precipitation.

Samples were treated with either 1 μg of RNase A (Roche) alone or with 1 μg of RNase A (Roche) and 1 U of *E. coli* RNase H (Roche) in a final volume of 10 μl containing 1x NEB buffer #2 at room temperature for 30 min. Nucleic acid material was subsequently extracted as described above.

All *in vitro* transcription reaction products were analyzed on 1% agarose gels run in 1x Tris–Borate–ethylenediaminetetraacetic acid (EDTA) buffer at 80 V for 3 h. Gels were subsequently stained with ethidium bromide (0.5 mg/ml) to allow visualization of total nucleic acid under ultraviolet (UV) light. For samples containing radioactive isotopes, gels were dried and exposed to X-ray film (Kodak BioMax XAR).

### Human cell extract treatment

HeLa S3 cell line was purchased from National Cell Culture Center, National Center for Research Resources, National Institutes of Health (Bethesda). The human SH-SY5Y neuroblastoma cells were cultured and neuronally differentiated with 10 μM all-*trans* retinoic acid (Sigma), with medium changes every 3 days as described (30,31). Whole cell extracts were prepared as described previously (32,33). Cell extracts prepared in this manner are functional in *in vitro* DNA replication, mismatch repair, excision repair (psoralens cross-links, cisplatin adducts, UV dimers, and pyrimidine dimers), double-strand break repair, homologous recombination, triplex-mediated recombination, processing of slipped-strand DNAs formed by CAG/CTG repeats (31,34–35), and are capable of inducing replication-mediated CAG/CTG expansions and contractions (26,36–37). R-loop templates prepared from *in vitro* transcription and RNase H treatments (described above) were incubated with ∼32 μg of extract in a 50 μl volume containing 100 μm each of deoxyadenosine triphosphate (dATP), dGTP, deoxythymidine triphosphate (dTTP), and deoxycytidine triphosphate (dCTP), guanosine triphosphate GTP, 200 μm each of uridine triphosphate (UTP), and cytidine triphosphate (CTP), 4 mm of adenosine triphosphate (ATP), 40 mm of creatine phosphate (Roche Molecular Biochemicals) and 100 μg/ml of creatine kinase (Roche Molecular Biochemicals). Reactions were carried out at 37°C for 4 h and subsequently stopped by adding 50 μl of stop solution (2 μg/μl proteinase K, 2% sodium dodecyl sulphate and 50 mm EDTA, pH 8.0) with further incubation for 60 min at 37°C. Nucleic acid material was subsequently extracted as described above. Samples were further purified using QIAquick enzyme clean-up kits as per manufacturer's instructions prior to transforming into bacteria for Stability of Trinucleotide Repeats by analysis of Individual Products (STRIP) analysis (see below). For *in vitro* DNA replication analysis, cell extract treatment was carried out in the same way as described above, but with the addition of 1 μg of SV40 T-antigen (Chimerx) and 0.1 Ci of [α-^32^P]dCTP.

### STRIP

The STRIP assay is described in detail previously (26,38). Briefly, products of human cell extract processing were transformed into *E. coli* XL1-MutS (Agilent) (Invitrogen). Individual bacterial colonies (each representing one processed template) were picked and cultured for a limited growth period (maximum of 6 h, 4–6 generations). During colony propagation, bacterial contribution was minimized for all clones by (i) having the (CTG)·(CAG) repeat in the stable orientation relative to the unidirectional bacterial ColE1 origin of replication; (ii) the limited colony growth time (≤6 h); and (iii) bacterial strain selection. Bacterial culture conditions including choice of bacterial strain have been thoroughly investigated in order to minimize background instability during the STRIP assay and have been described in detail previously (26,38). Miniprep DNA was analyzed for changes in repeat length by analysis of the repeat-containing fragment on 4% polyacrylamide gels. Repeat lengths (CAG/CTG) were classed into one of three categories: ‘<79 repeats’, ‘79 repeats’ and ‘>79 repeats’ representing contraction, stable and expansion products, respectively. Similar procedures were used for the *C9orf72* clones. The magnitudes of repeat length changes were determined by electrophoretic sizing of the repeat-containing fragments on 4% polyacrylamide gels relative to the starting length material and a known set of size markers.

### Electron microscopy

RNase A and RNase H-treated transcription reaction products were de-proteinized and analyzed by electron microscopy (EM) as described previously (3). Briefly, binding reactions with bacterial single-strand binding protein (SSB) were carried out in a 50 μl reaction mixture containing 8 mM NaCl, 20 mM HEPES (hydroxy ethyl piperazine ethanesulfonic acid) (pH 7.5) and 300 ng SSB for 10 min at room temperature. Complexes were fixed with 0.6% glutaraldehyde (v/v) for 10 min at room temperature, followed by filtration through a 2-ml column of Bio-Gel A5m (Bio-Rad) to remove excess glutaraldehyde and free proteins. Fractions containing DNA–SSB protein complexes were prepared for EM. Briefly, the indicated SSB–DNA complexes were mixed in a buffer containing 2 mM spermidine, adsorbed to glow-charged carbon-coated grids, washed with a water/graded ethanol series and rotary shadow cast with tungsten. Samples were examined using a Philips 420 electron microscope. Micrographs are shown in reverse contrast.

## RESULTS

### R-loops can be processed by human cell extracts

**Figure 1.**
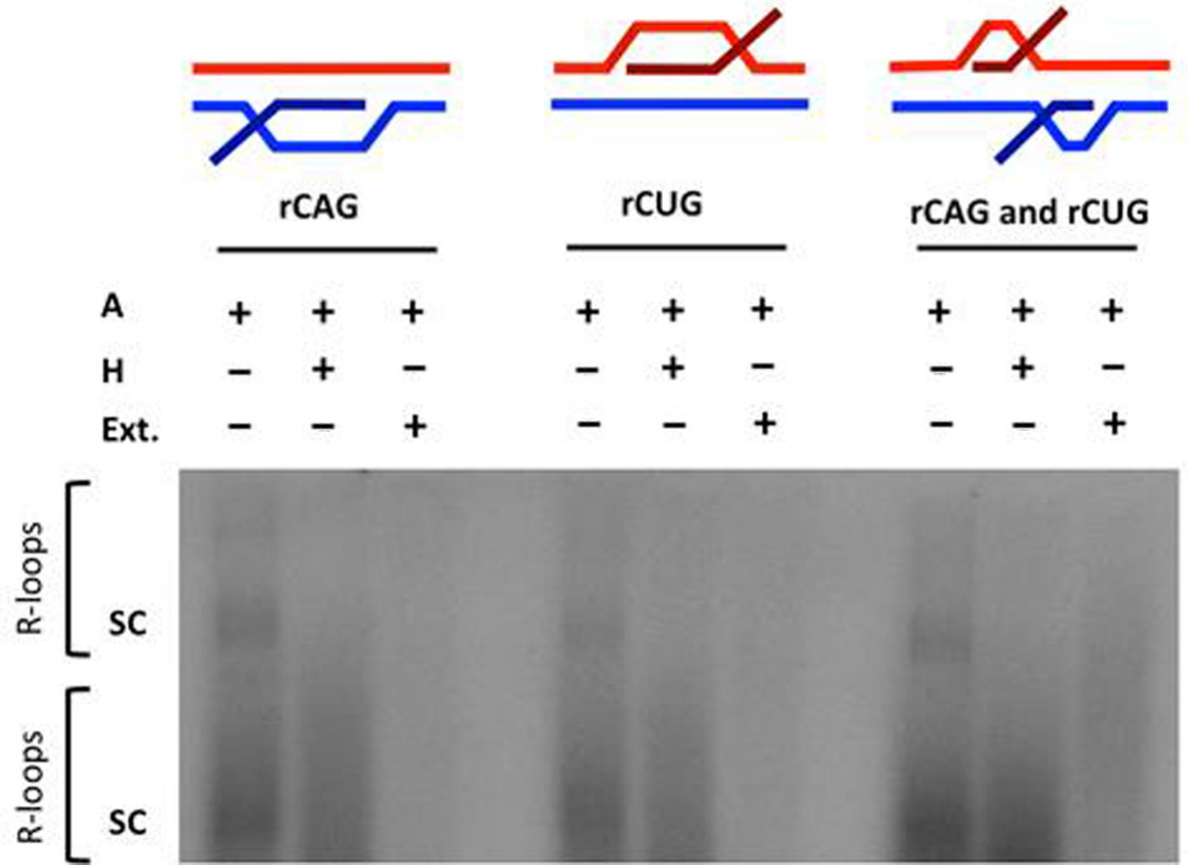
R-loop processing by human cell extract. *In vitro* transcription of a (CAG)_79_·(CTG)_79_ repeat-containing plasmid with [α-^32^P]rCTP was performed followed by RNase A treatment (to cleave single-stranded RNA); labeled ‘A’ or RNase H treatment (to also cleave RNA:DNA hybrids of the R-loop); labeled ‘H’ or human cell extract treatment; labeled ‘Ext.’ as indicated. The configuration of the R-loop generated is schematically represented above the gel. Autoradiographic signal in the gel represents R-loop formation. The position of supercoiled plasmid in dimer and monomer form is indicated by ‘sc’ where the top ‘sc’ represents linked dimers and bottom ‘sc’ represents monomers.

**Figure 2.**
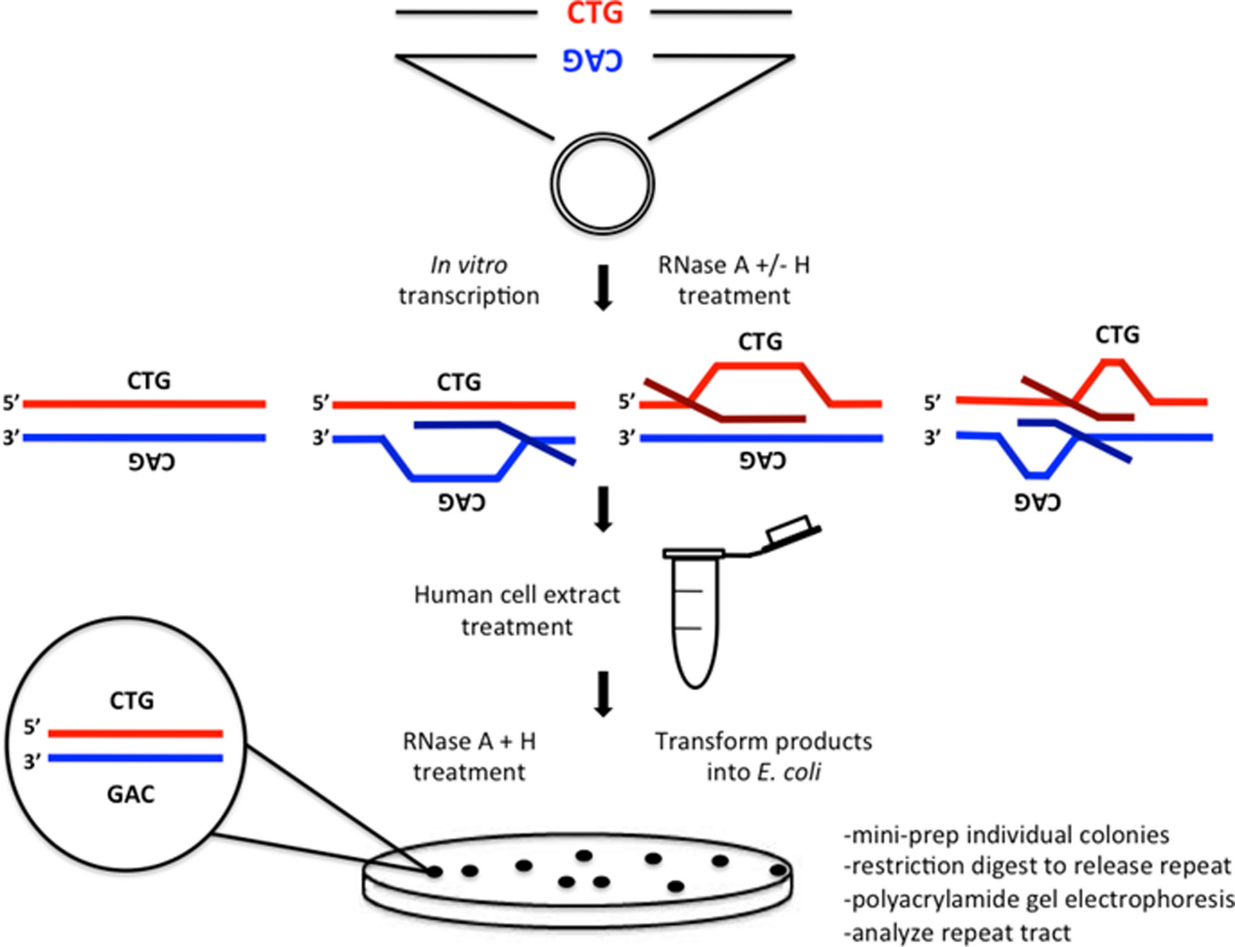
R-loop instability assay. Plasmids bearing an expanded (CAG)_79_·(CTG)_79_ repeat tract were in vitro transcribed and treated with the appropriate RNase to produce R-loop templates of each configuration as schematically depicted. R-loop templates were subsequently treated with human (HeLa) cell extract to allow processing to occur. Nucleic acid products were extracted and subjected to a final RNase A+H treatment to remove any residual R-loop products and transformed into E. coli bacteria and plated overnight. Individual colonies were picked (representing individual products of R-loop processing) and minimally cultured (see Materials and Methods). DNA was then extracted and restriction digested to release the repeat tract. Products were electrophoresed alongside known size markers to determine repeat length.

**Figure 3.**
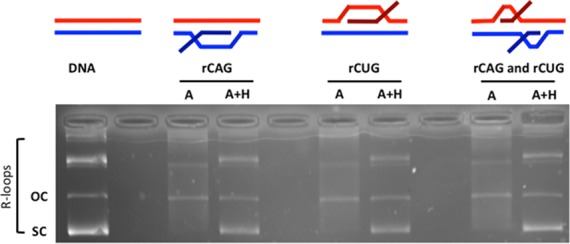
R-loop formation in (CAG)_79_·(CTG)_79_ templates. Templates were in vitro transcribed with T3 and/or T7 RNA polymerases and treated with RNase A (which digests single-stranded RNA) to form each R-loop configuration indicated schematically above the gel. The presence of R-loops forces the plasmid into a more open configuration, thus reducing electrophoretic migration within the gel. Treatment of the R-loop with RNase H cleaves RNA that is base-paired to DNA (the RNA:DNA hybrid) and thus collapses the R-loop, returning DNA to supercoiled form. The position of supercoiled plasmid is indicated as ‘sc’ and open circular plasmid as ‘oc’. Products above these are catenated multimers, which also form R-loops.

To investigate the role of R-loops in (CAG)·(CTG) repeat instability, we utilized human whole cell extracts (HeLa) that are proficient in *in vitro* DNA repair in an *in vitro* processing assay which we modified from previous studies of repeat instability in the lab (26,31,34–35). To assess the status of the R-loop following human cell extract treatment, we first generated R-loops through *in vitro* transcription with [α-^32^P]rCTP-incorporation into the nascent RNA. Following treatment with RNase A (a ribonuclease that digests unbound, single-stranded RNA), there was a resistant autoradiographic signal consistent with R-loop formation as demonstrated previously (3) (Figure 1). Treatment of these products with both RNase A and RNase H (a ribonuclease that specifically degrades RNA that is bound to DNA as would occur in an R-loop) led to a reduction in the RNase A-resistant autoradiographic signal in the supercoiled forms of the DNA template consistent with digestion of R-loop material as demonstrated previously (3) (Figure 1; sc). However, we saw some signal persists indicating incomplete digestion of the products, which is consistent with the inability of RNase H to recognize and cleave RNA in an RNA:DNA hybrid shorter than four base pairs in length (39) as well as the extremely high sensitivity of autoradiography relative to ethidium bromide staining (compare Figures 1–3; A and A+H treatments). While R-loops are known to be favorably retained in supercoiled state (3), interestingly, this material that is partially resistant to the purified RNase H, is more stable in the monomeric rather than the concatameric forms. Upon treatment of the R-loops with HeLa cell extract, the autoradiographic RNA signal was largely reduced, suggesting that the HeLa extract is able to more completely process R-loop substrates, likely due to the presence of other factors (Figure 1).

To test whether the R-loops were acting as initiation sites for replication, where the RNA portion may serve as a primer for initiation by the HeLa cell extract, we analyzed dNTP radioincorporation into the R-loop-containing templates in the presence of HeLa cell extract (Supplementary Figure S1). Radioincorporation was compared to templates that did not contain R-loops, but were incubated with HeLa cell extract in the presence of T-antigen, which is able to initiate *in vitro* DNA replication from an SV40 origin of replication present in our repeat-containing templates (Supplementary Figure S1). We only observed high levels of dNTP radioincorporation that was *Dpn*I-resistant, indicative of DNA replication, in the presence of T-antigen and not when R-loop templates were incubated with HeLa cell extract (Supplementary Figure S1). Thus, the R-loops do not act as an initiation site for DNA replication in our system.

### Establishing a (CAG)·(CTG) repeat R-loop processing system

To assess whether R-loop processing might alter the DNA lengths of the (CAG)·(CTG) repeat tracts, we carried out a modified version of our previously reported STRIP protocol (Figure 2) (26,36–38). Essentially, the repeat tract lengths of single molecules were assessed in R-loop preparations that had been processed by HeLa cell extracts and compared to the same DNA templates devoid of R-loops that had also been processed by HeLa cell extracts. R-loop substrates for HeLa cell extract processing were generated through *in vitro* transcription across the repeat, initiated by T3 and/or T7 phage RNA polymerases, whose promoters flank the repeats, as previously described (Figure 3) (3). Single-stranded RNA was eliminated from R-loop preparations by treating transcription products with RNase A. Controls where the RNA portion was eliminated from the R-loop, were prepared by treatment with both RNase A+H (Figure 3). This preparation potentially contains aberrant S-DNAs upon R-loop removal, as noted above (see also (3)). A DNA template devoid of R-loops that had not been transcribed served as a non-R-loop and non-S-DNA control (Figure 3). These substrates were all treated with HeLa cell extract and repeat lengths were analyzed by STRIP (Figure 2).

### Processing of double-R-loops generates increased instability

**Figure 4.**
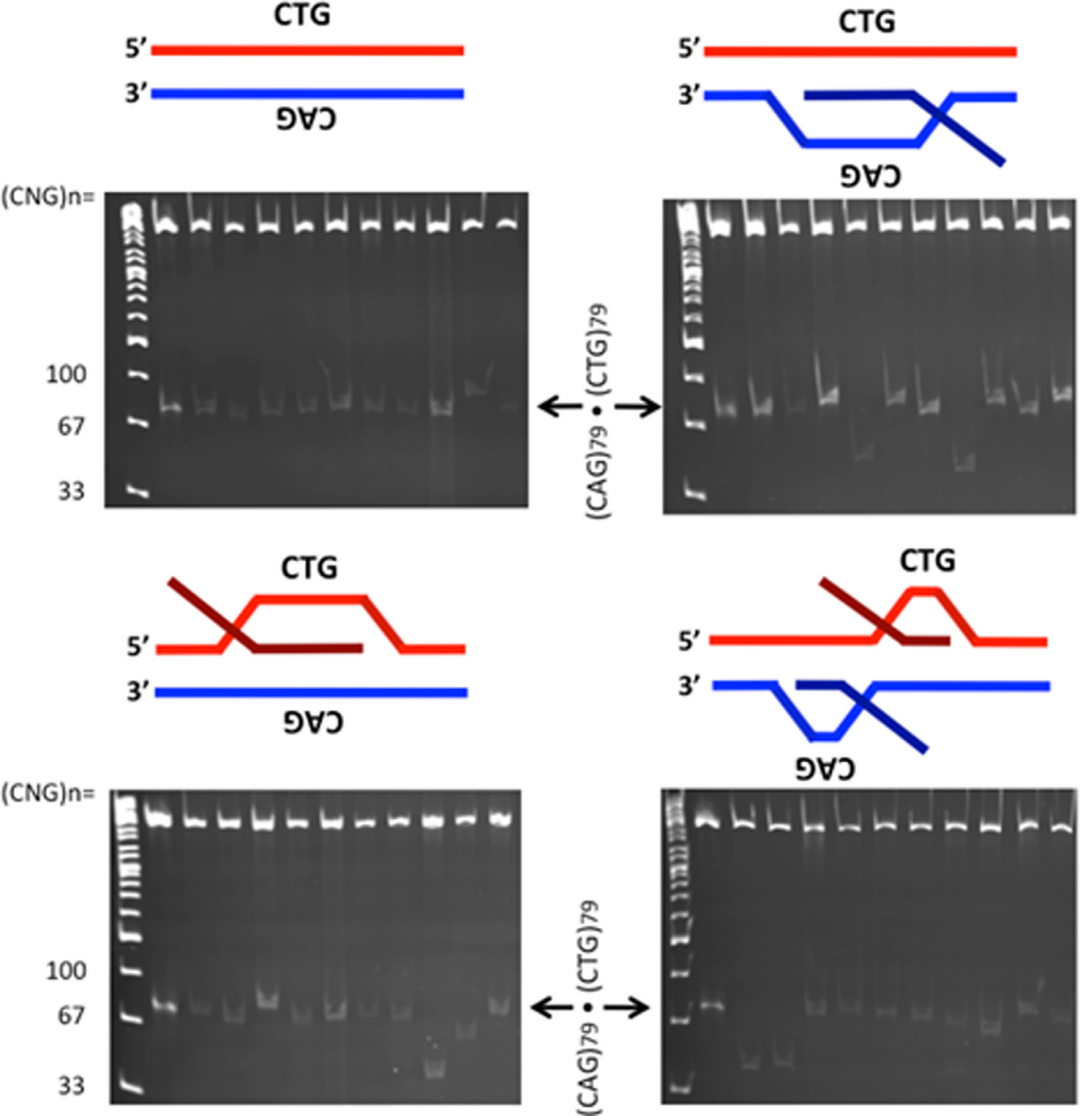
Products of R-loop processing by gel analysis. R-loop templates were formed by *in vitro* transcription; the DNA control template was not transcribed, while the other templates were transcribed and treated with RNase A to remove single-stranded RNA but retain the R-loop configuration as indicated schematically above each gel. These templates were treated with HeLa cell extract. Products of HeLa extract processing were extracted, treated with RNase A+H to remove residual R-loops and transformed into *E. coli.* Following minimal culturing, products of each R-loop configuration were resolved on polyacrylamide gels alongside known size markers. The product immediately adjacent to the ladder lane in each gel (indicated by arrow) is the untranscribed, unprocessed parental DNA template that serves as a size marker containing 79 (CAG)·(CTG) repeats.

Varying levels of repeat instability were evident by electrophoretic resolution of repeat-containing fragments from individual molecules, exhibited by slower-migrating repeat-containing fragments (representing repeat tract expansions) as well as by faster-migrating fragments (representing repeat tract contractions) (Figure 4, each lane represents one individual molecule following HeLa extract treatment). The levels of instability appeared to depend upon the substrate configuration. An aliquot of the starting parental template with (CAG)_79_·(CTG)_79_ repeats that had not been transcribed nor subjected to cell extract treatment or STRIP, was loaded in the first lane of each gel to serve as a marker to compare products of instability against (Figure 4, indicated by arrow). The non-transcribed DNA template that was treated with human cell extract and subjected to STRIP served as the cell extract processing control to assess basal levels of length heterogeneity present in the starting material (instability during preparation in bacteria), as well as any instability that may be incurred by exposure of the fully-paired DNA repeat to the HeLa extract (Figure 4). Some level of length heterogeneity is expected for the DNA template due to its unstable length of 79 repeat units, resulting from processing of endogenous DNA damage (potentially including single strand breaks, oxidative damage, nucleotide mismatches, etc.) by human cell extract, repeat length heterogeneity present in the starting plasmid, as well as from bacterial culturing during the STRIP procedure (26,38). The tract length heterogeneity in this starting template serves as the background level of tract length instability above which any potential R-loop processing instability must rise.

**Figure 5.**
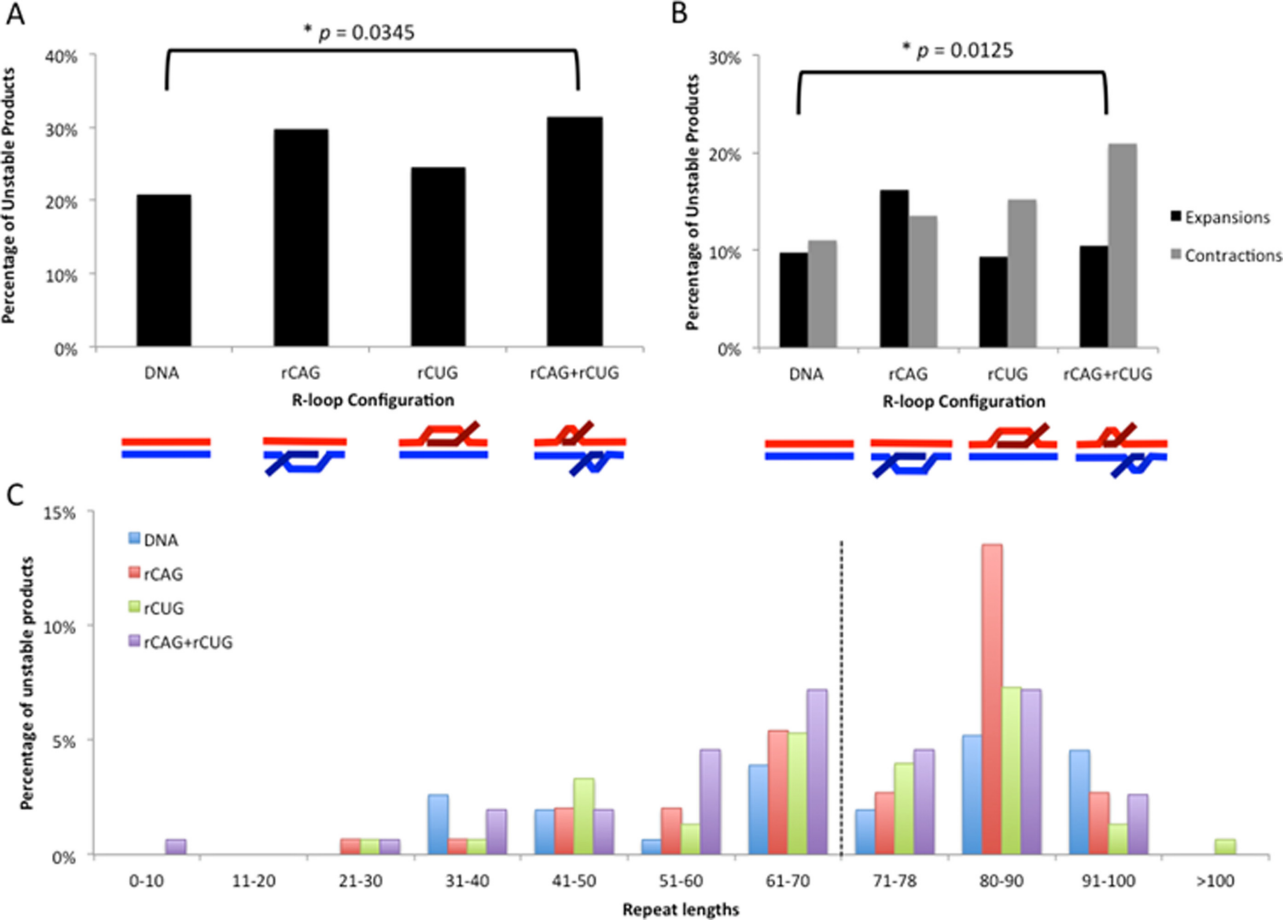
Instability analysis of products from R-loop processing by human cell extract. (**A**) Percentage of total unstable products following processing. Products were characterized as either stable (having 79 repeats) or unstable (having fewer than or greater than 79 repeats), based on electrophoretic migration and plotted. Data are derived from three independent *in vitro* transcription and human cell extract processing reactions with ∼150 colonies (∼50 colonies per replicate) representing 150 individual products of cell extract treatment analyzed for each R-loop configuration. Individual experiments were compared with each other within a triplicate using the *χ*^2^ test to ensure there were no significant differences between experiments and then data were pooled for each experimental condition. Specific colony numbers are as follows: DNA-154, rCAG-148, rCUG-151, rCAG+rCUG-153. Products of R-loop processing were compared to the DNA control processing products using the *χ*^2^ test. (**B**) Percentage of contractions and expansions from processing. Unstable products were further separated into contractions (fewer than 79 repeats) and expansions (greater than 79 repeats) and plotted. The distribution of contractions and expansions were compared between R-loop products and DNA control products using the *χ*^2^ test. (**C**) Distribution of unstable products of R-loop processing. Sizes were estimated for each unstable product of processing from electrophoretic migration position relative to known size markers as previously described (26) and plotted. Only unstable products are shown; the stable repeat size of 79 is indicated by the dashed vertical line.

The various R-loop substrates generated increased levels of repeat instability following HeLa extract treatment relative to the control DNA template (Figure 4 shows representative gels). We quantified the differences in instability resulting from each R-loop processing reaction by assessing the repeat sizes of a large set of single molecules, compared to known size markers. Products were categorized as ‘stable’ (having 79 repeats), ‘contractions’ (having fewer than 79 repeats) or ‘expansions’ (having greater than 79 repeats) (Figure 5). There was a significant increase in instability from processing of R-loops formed by simultaneous convergent transcription relative to the DNA control template that had not been transcribed (Figure 5A, *P* = 0.0345 by *χ*^2^ analysis). There was a modest but consistent increase in instability arising from HeLa cell extract processing of R-loops from either direction alone, but the differences were not significant compared to DNA. It is noteworthy that instability arising from rCAG R-loop processing was consistently higher than instability arising from rCUG R-loop processing (Figure 5A). This observation reflects upon previous findings that rCAG R-loops exhibit increased formation/stability than rCUG R-loops (3,11).

To ensure that the differences in instability we observed were a result of HeLa extract processing and not from culturing in bacteria, we performed STRIP analysis as described above, but transformed the products of *in vitro* transcription following RNase A+H treatment into the bacteria without HeLa cell extract treatment (Supplementary Figure S2). There were no significant differences in instability between DNA control and R-loop templates without HeLa extract treatment (Supplementary Figure S2).

### Distribution of products from R-loop processing

Repeat instability can vary both in mutation direction (expansion versus contraction) and magnitude of change. The unstable products of HeLa cell extract processing from the various R-loop configurations showed increases in expansions and contractions compared to the DNA control (Figure 5B). Processing of rCAG+rCUG double-R-loops resulted in a significant increase in contractions compared to control DNA (Figure 5B; *P* = 0.0125 by *χ*^2^ analysis). Processing of rCUG and rCAG+rCUG double-R-loops yielded more contractions than expansions (Figure 5B). On the other hand, processing of rCAG R-loops yielded more expansions than contractions (Figure 5B). Thus, R-loop configuration can influence the direction of instability (expansion versus contraction).

The magnitudes of repeat length changes for each R-loop processing are plotted in Figure 5C. Most of the mutant products showed small changes, relative to the starting template size distribution in the (CAG)_79_·(CTG)_79_ preparation. There were fewer products with repeat numbers increasing or decreasing from this starting parental length (Figure 5C). The products from DNA control processing reactions displayed a range of repeat sizes from 31 to 100 units. The range of products in the R-loop-processed reactions was broader, from as few as 8 repeats (rCAG+rCUG processing) to as many as 127 repeats (rCUG processing). Repeat sizes of all products from human cell extract processing are listed in Supplementary Table S1.

### Neuron-like extracts yield instability through double-R-loop processing

**Figure 6.**
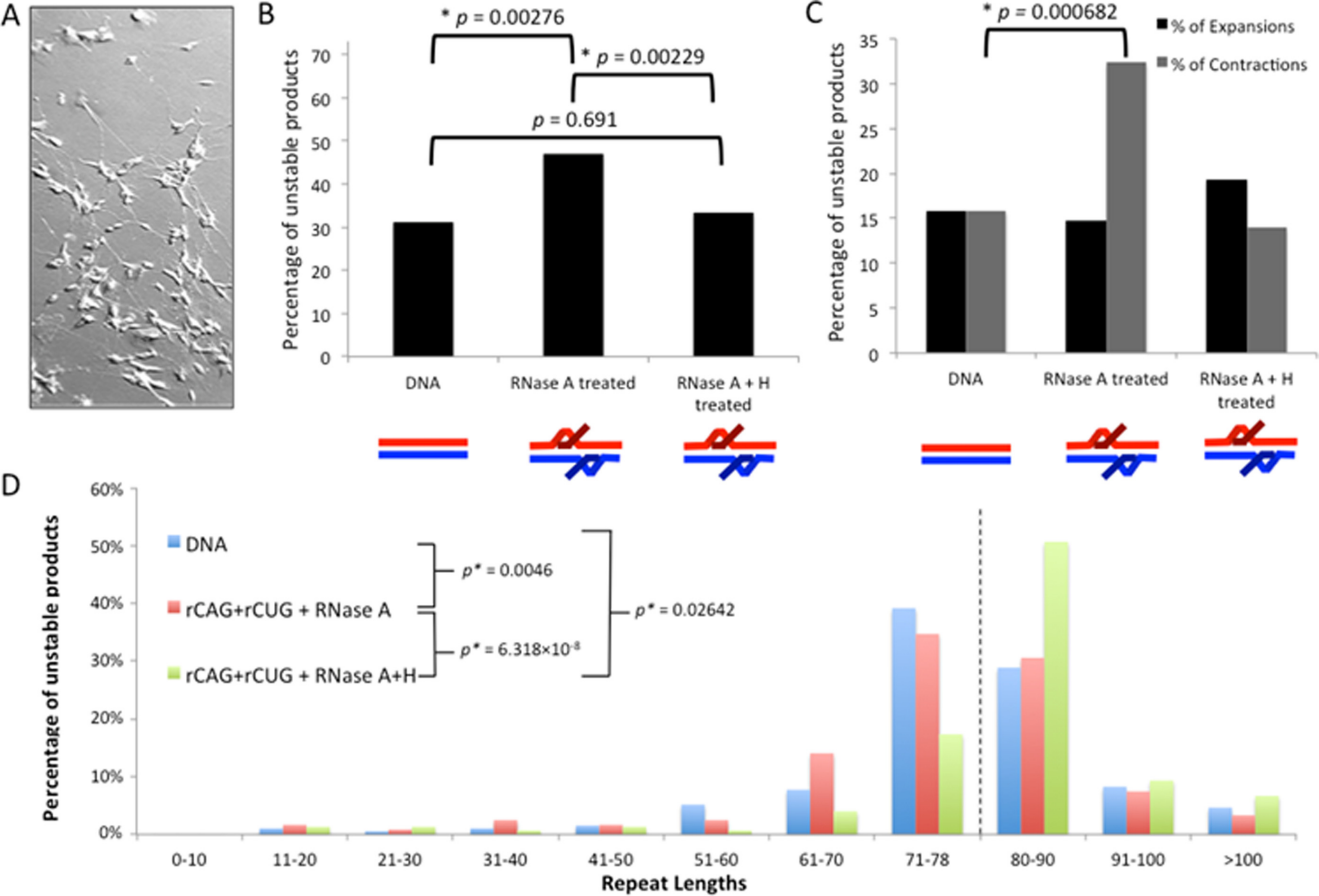
Instability analysis of products from double-R-loop processing by neuron cell extract. (**A**) Retinoic acid-differentiated SH-SY5Y cells. (**B**) Percentage of total unstable products following processing. Products were characterized as either stable (having 79 repeats) or unstable (having fewer than or greater than 79 repeats), based on electrophoretic migration and plotted. Data are derived from three independent *in vitro* transcription and retinoic acid human SH-SY5Y cell extract processing reactions with ∼150 colonies (∼50 colonies per replicate) representing 150 individual products of cell extract treatment analyzed for each R-loop configuration. Individual experiments were compared with each other within a triplicate using the *χ*^2^ test to ensure there were no significant differences between experiments and then data were pooled for each experimental condition. Specific colony numbers are as follows: DNA-194; rCAG+rCUG+RNase A-121; rCAG+rCUG+RNase A+H-149. Products of R-loop processing were compared to the DNA control processing products using the *χ*^2^ test. (**C**) Percentage of contractions and expansions from processing. Unstable products were further separated into contractions (fewer than 79 repeats) and expansions (greater than 79 repeats) and plotted. The distribution of contractions and expansions were compared between R-loop products and DNA control products using the *χ*^2^ test. (**D**) Distribution of unstable products of R-loop processing. Sizes were estimated for each unstable product of processing from electrophoretic migration position relative to known size markers as previously described (26) and plotted. Only unstable products are shown; the stable repeat size of 79 is indicated by the dashed vertical line.

Somatic expansions occur in the central nervous system of patients suffering CAG/CTG expansions and mouse models of these diseases (14,40). These ongoing mutations may exacerbate age-of-onset, disease severity and progression. Neural differentiation is associated with changes in repair capacity (41). Using extracts of the non-proliferating human SH-SY5Y cells differentiated into neuron-like cells (Figure 6A) (30,31), we assessed whether R-loop processing could alter repeat lengths. The products of SH-SY5Y extract-processed double-R-loops showed moderate, yet significantly increased levels of instability compared to the mock-treated DNA (Figure 6B, *P* = 0.00276, by *χ*^2^ analysis). Processing of double-R-loops that have had the RNA component removed (RNase A+H) did not alter levels of instability compared to the control DNA, but was significantly different from processing of the intact double-R-loop (Figure 6A, *P* = 0.691, *P* = 0.00229, respectively by *χ*^2^ analysis). Processing of intact double-R-loops resulted in a significant increase in contractions compared to control DNA (Figure 6C; *P* = 0.000682052 by *χ*^2^ analysis). Thus, double-R-loop processing by a neuron-like cell extract can lead to repeat instability.

The magnitudes of repeat length changes for double-R-loop processing are plotted in Figure 6D, see also Supplementary Table S3. Most of the mutant products showed small changes, relative to the starting template size distribution in the (CAG)_79_·(CTG)_79_ preparation. The products from DNA control SH-SY5Y-processing reactions displayed a wide range of repeat sizes, from 20 to 131 repeat units, with a distribution similar to the starting material. The size distribution of the DNA products of SH-SY5Y-treated double-R-loops was significantly different prior to removal of the RNA portion (RNase A, *P* = 0.00046, Wilcoxon signed rank test), and to a lesser degree following its removal (RNase A+H, *P* = 0.02642, Wilcoxon signed rank test). These results further support the suggestion double-R-loop processing by a neuron-like cell extract can lead to repeat instability.

### RNase H treatment partially suppresses increased instability from R-loop processing

**Figure 7.**
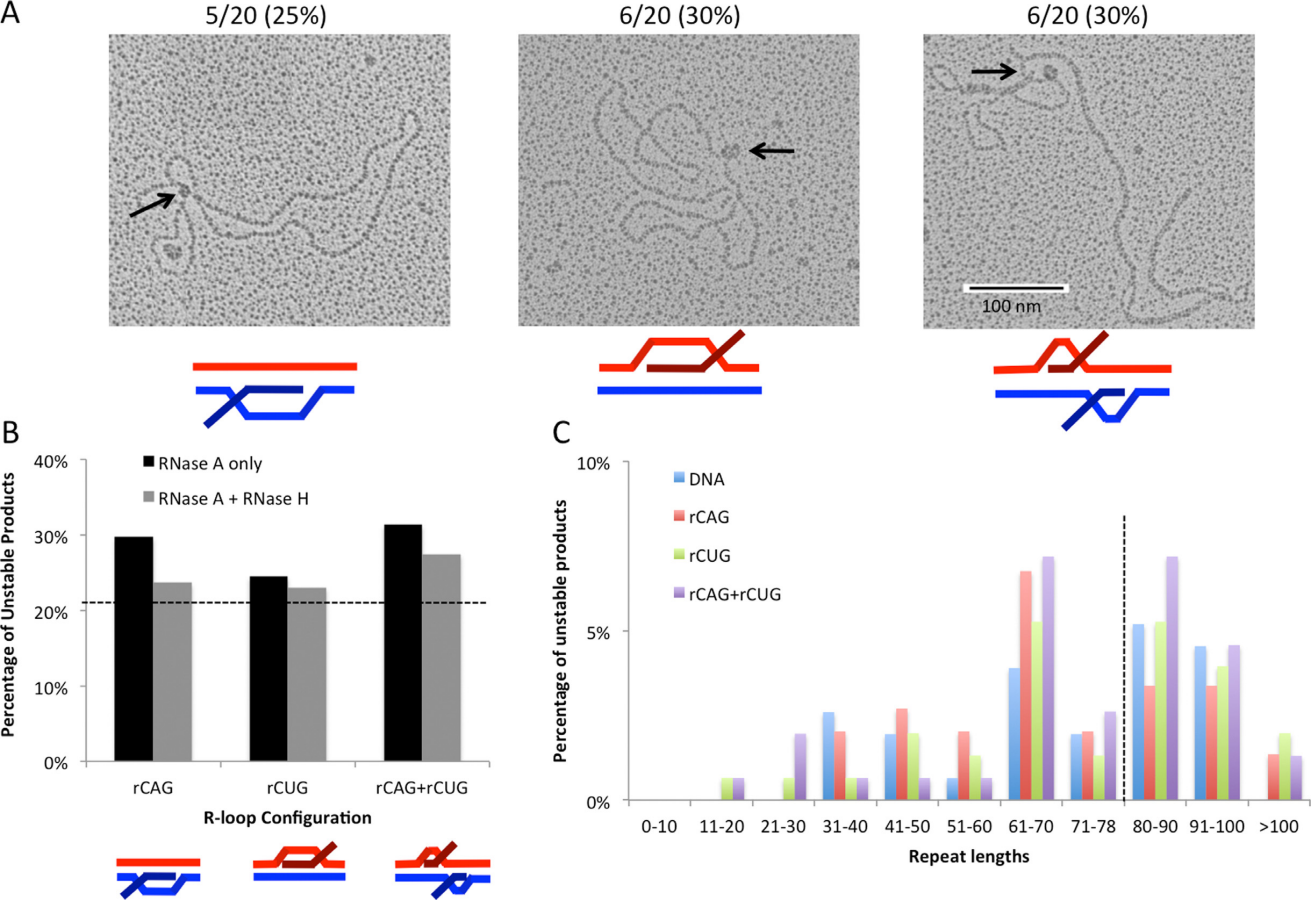
Instability analysis following R-loop removal. (**A**) Detection of S-DNA structures following R-loop removal with RNase H. DNA templates were transcribed to generate r(CAG), r(CUG) or r(CAG)+r(CUG) R-loops and then treated with RNase A and RNase H to remove all single-stranded RNA and RNA:DNA hybrids. EM was performed on individual molecules in the presence of bacterial SSB to detect unpaired DNA strands that exist in S-DNA structures (see ‘Materials and Methods’). A total of 20 molecules were analyzed for each sample type. DNA controls that were not transcribed contained 2/20 (10%) molecules bound by SSB. The number of samples bound by SSB at a single position following R-loop removal is indicated above each image and expressed as a percentage. (**B**) Percentage of unstable products following processing of RNase H-treated R-loops. R-loop products of each configuration were treated with RNase H prior to cell extract processing and assessed for instability through STRIP analysis as in (A). Products from (A) (RNase A only) were compared to RNase H-treated R-loops (RNase A+H) using the *χ*^2^ test. Data for RNase H-treated R-loop processing are derived from three independent *in vitro* transcription and human cell extract processing reactions with ∼150 colonies representing 150 individual products of cell extract treatment for each RNase H-treated R-loop configuration. Specific colony numbers are as follows: rCAG-148, rCUG-152, rCAG+rCUG-153. Dashed line indicates DNA control level of instability (21%) for comparison. (**C**) Distribution of unstable products of HeLa extract processing following RNase-H-mediated R-loop removal. Sizes were determined for each unstable product of processing from electrophoretic migration position relative to known size markers as previously described (26) and plotted. Only unstable products are shown; the stable repeat size of 79 is indicated by the dashed vertical line.

Removal of the RNA component of the R-loop can alter the structure of the remaining DNA template, such that homo-duplex S-DNAs may arise following out-of-register reannealing of the complementary DNA strands (3). Using EM, we analyzed individual molecules following R-loop removal with RNase H (Figure 7A). There was an increase in the number of molecules bound by SSB following R-loop removal compared to DNA control samples that were not transcribed (Figure 7A). Levels of S-DNAs were similar for each R-loop configuration. The binding of SSB to DNA templates following R-loop removal supports the formation of S-DNA structures that are unpaired from their complementary DNA strand and contain regions of single-strandedness detectable by SSB binding. As previously reported, slip-out sizes were too small to detect in the absence of SSB (42).

To further support the formation of S-DNA structures following RNase H removal, templates were additionally treated with mung bean nuclease, which degrades single-stranded DNA, as would be present in slipped-structures following RNA:DNA hybrid removal with RNase H (Supplementary Figure S3). Visualization by EM revealed that there was an increase in the number of molecules bound by SSB following R-loop removal with RNase H compared to a DNA template control and a reduction in the number of molecules bound by SSB following mung bean nuclease treatment as would be expected due to the formation of S-DNA structures following R-loop removal with RNase H (Supplementary Figure S3).

We next assessed whether the DNA remaining after RNA elimination from the R-loop can lead to instability through processing by HeLa cell extracts. We analyzed the instability arising from R-loops that were treated with RNase H prior to treatment with HeLa cell extract (Figure 7B). For each R-loop configuration, treatment with RNase H prior to HeLa extract treatment consistently reduced the overall instability (Figure 7B). Interestingly, the RNase H-induced reduction in instability was only partial relative to DNA control and instability was not significantly less than R-loop preparations that were not treated with RNase H prior to processing by HeLa extract (Figure 7B). Furthermore, analysis of the individual unstable products resulting from RNase H-treated samples revealed a similar distribution as observed with processing of R-loop samples without RNase H-treatment (compare Figures 5C–7C, and Supplementary Tables S1–S2). These products also showed a broader length distribution than products from the DNA control, which did not contain repeats shorter than 31 units or longer than 100 units (Figure 7C). These observations were most notable for double-R-loop processing following R-loop removal with RNase H (Figure 7). Thus, RNA degradation from the R-loop partially suppresses instability evident from the modest but consistent decrease in instability upon RNase H treatment; however, R-loop removal with RNase H still triggers instability, which may occur through the formation of S-DNA structures (as evidenced by EM) through misaligned reannealing of the repeat tract.

### R-loop formation in *C9orf72* repeats, processing and instability

**Figure 8.**
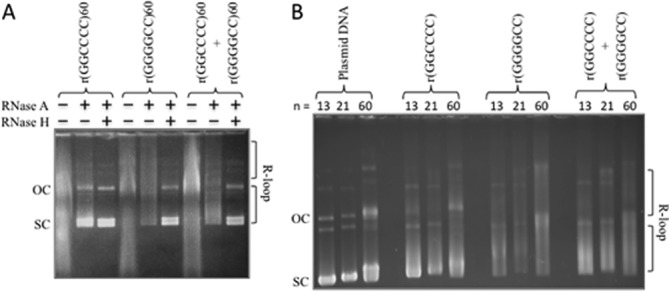
*C9orf72* repeat forms R-loops and double-R-loops. (**A**) Templates with (GGGGCC)_60_·(GGCCCC)_60_ were *in vitro* transcribed with T3 and/or T7 RNA polymerases and treated with RNase A (which digests single-stranded RNA) to form each R-loop configuration indicated schematically above the gel. The presence of R-loops forces the plasmid into a more open configuration, thus reducing electrophoretic migration within the gel. Treatment of the R-loop with RNase H cleaves RNA that is base-paired to DNA (the RNA:DNA hybrid) and thus collapses the R-loop, returning DNA to supercoiled form. The slower migrating products above these are catenated multimers, which also form R-loops. (**B**) Templates with 13, 21 or 60 *C9orf72* repeats were transcribed as in panel (A) to reveal single and double-R-loop formation.

To assess whether R-loops may form at the expanded *C9orf72* (GGGGCC)*_n_*·(GGCCCC)*_n_* repeats, we used a series of clones with tract lengths ranging from 13 to 60 repeats. Transcription of a tract with 60 repeats in either direction producing r(GGCCCC) or r(GGGGCC) leads to R-loop formation, resistant to RNase A and sensitive to RNase H (Figure 8A), consistent with R-loop formation (28,29). Transcription through the C-rich template strand produced slightly more hybrids compared to the G-rich template strand. Simultaneous transcription across both strands also led to double-R-loops. To assess a potential length effect, we used tracts with 13, 21 and 60 repeats; these too formed R-loops and double-R-loops, with little variation in amounts of R-loops formed (Figure 8B). Thus, this ALS/FTD disease-associated hexanucleotide repeat tract can also form biophysically stable single- and double-R-loop structures.

**Figure 9.**
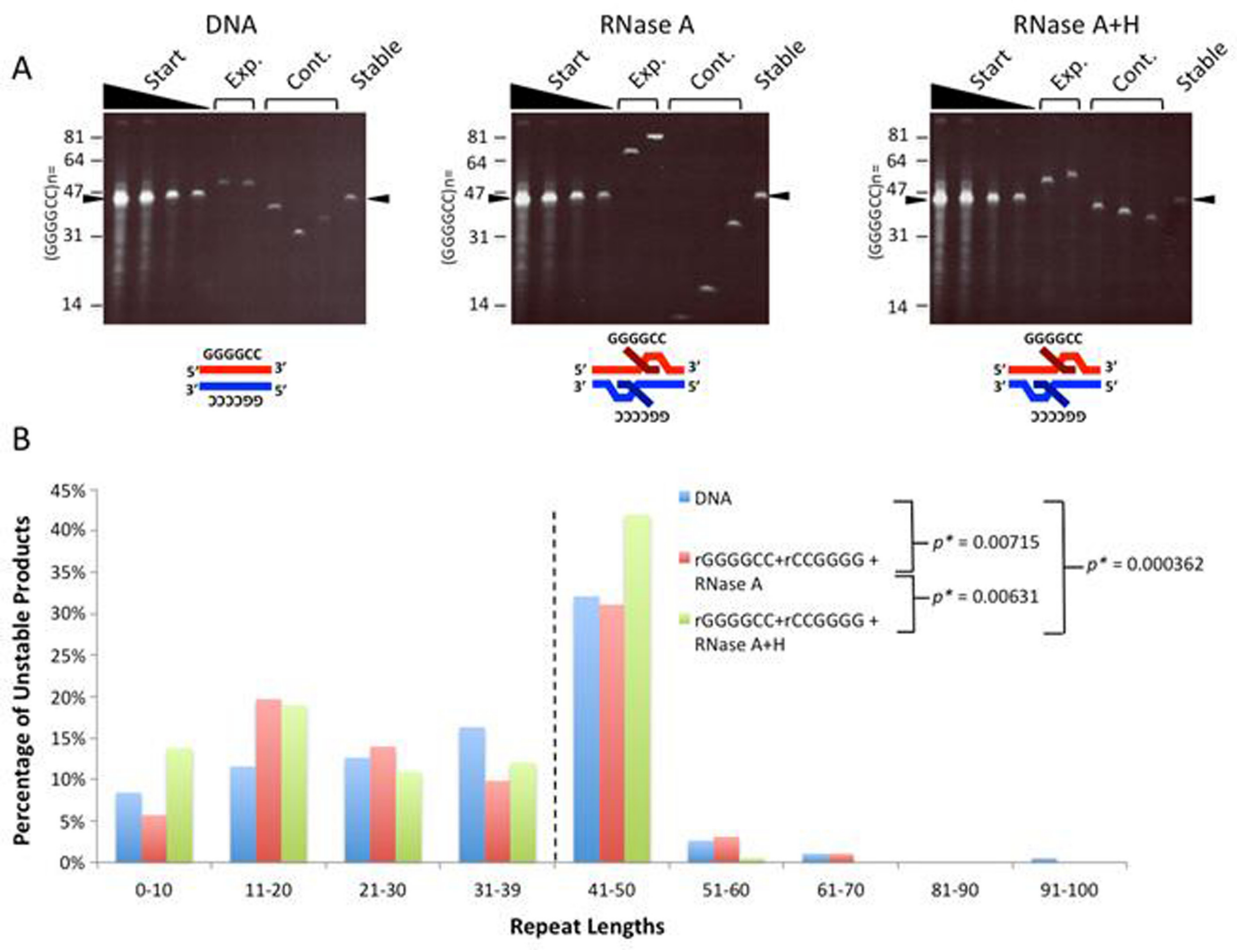
*C9orf72* double-R-loops when processed by neuron cell extracts lead to instability. (**A**) Products of double-R-loop processing by gel analysis. DNA control template with (GGGGCC)_40_·(GGCCCC)_40_ was not transcribed, while the other templates were transcribed and treated with RNase A to remove single-stranded RNA, but retain the R-loop configuration as indicated schematically above each gel. These templates were treated with SH-SY5Y cell extract. Products of SH-SY5Y extract processing were extracted, treated with RNase A+H to remove residual R-loops and transformed into *E. coli.* Following minimal culturing, products of each R-loop configuration were resolved on polyacrylamide gels alongside known size markers. For each gel, the first four lanes show a titration of DNA concentration of the starting DNA, to reveal repeat length heterogeneity. A sample of products with lengths larger, smaller or similar to the starting lengths are shown. Specific colony numbers are as follows: DNA-168; rGGGGCC+rGGCCCC+RNase A-167; rGGGGGCC+rGGCCCC+RNase A+H-157. The arrowhead indicates the size of the major starting length of 40 repeats. (**B**) Distribution of unstable products of R-loop processing. Sizes were estimated for each unstable product of processing from electrophoretic migration position relative to known size markers as previously described (26) and plotted. Only unstable products are shown; the starting major repeat size of 40 is indicated by the dashed vertical line.

To test whether processing of these *C9orf72* R-loops might alter repeat tract lengths, we prepared double-R-loops from a plasmid with 40 repeats, treated these with RNase A or RNase A+H and subjected these to SH-SY5Y cell extracts. These repeats were extremely unstable in bacterial preparations, showing extremely high levels of unavoidable length heterogeneity, all of which are contractions of the starting length (Figure 9A, see titrated DNA concentration in first four lanes of each gel). Due to this high level of variation in the starting preparation, we assessed length distributions as a measure of instability. A sample of the individual products is shown in Figure 9A (see also Supplementary Table S4). The products from DNA control SH-SY5Y-processing reactions displayed a broad range of repeat sizes from 4 to 64 units, similar to the starting material. Products in the R-loop-processed reactions ranged from 5 to 69 repeats. However, the size distribution of the DNA products of SH-SY5Y-treated double-R-loops was significantly different prior to removal of the RNA portion [DNA versus RNase A, *P* = 0.00715, confidence interval (CI) 95%, Wilcoxon signed rank test]. Interestingly, following removal of the RNA from the double-R-loop, treatment with SH-SY5Y extracts led to a significantly different size distribution of the DNA products (DNA versus RNase A+H, *P* = 0.000362, CI 95%, Wilcoxon signed rank test), which differed significantly from the intact double-R-loop (RNase A+H versus RNase A, *P* = 0.00631, CI 95%, Wilcoxon signed rank test). These results provide the first possible mechanism through which the *C9orf72* repeats may be unstable and further support the suggestion double-R-loop processing by a neuron-like cell extract can lead to repeat instability. These results also suggest that processing of a *C9orf72* double-R-loop that has had its RNA component removed, can also lead to instability.

## DISCUSSION

In this study, we have developed a repeat R-loop processing assay to assess how such processing might contribute to repeat instability. We show that R-loops generated by *in vitro* transcription of an expanded (CAG)_79_·(CTG)_79_ DNA repeat tract as well as an expanded (GGGGCC)_40_·(GGCCCC)_40_ can be processed by human cell extract to increase instability of the repeat tract. Specifically, we demonstrate that (i) there is a significant increase in instability (*P* <0.05) following double-R-loop processing, but only a modest increase following single-R-loop processing, which was not found to be statistically significant; (ii) the range of repeat length changes is broader as a result of R-loop processing compared to DNA controls; (iii) RNase H treatment only partially suppresses R-loop-mediated instability; (iv) slipped-DNAs can be formed following degradation of the RNA from R-loops; and (v) double-R-loop processing by extracts of a non-proliferating neuronally-differentiated cell line can also lead to instability.

The mechanism through which R-loops cause disease-associated repeat instability is poorly understood. Some studies on the role of R-loops in genome-wide instability support a mechanism involving DNA replication fork stalling in the presence of R-loops leading to aberrant recombination (43,44). In the case of (CAG)·(CTG) instability however, transcription is able to generate instability independent of DNA replication (19), suggesting direct engagement of repair factors by secondary structures generated during or following transcription (3,11). This model of aberrant DNA repair is consistent with instability arising in post-mitotic cells like neurons, which in many diseases display high levels of repeat instability (14). However, the idea that aberrant R-loop processing could lead to repeat instability had not been previously tested. In this study, we demonstrate that transcriptionally formed R-loop structures can directly trigger instability through human cell extract processing. Thus, genome maintenance alone may account for R-loop-mediated instability in non-replicating tissues. However, it should be noted that although not required, DNA replication may further exacerbate R-loop-associated (CAG)·(CTG) instability, e.g. if transcription and replication machinery were to collide at expanded (CAG)·(CTG) repeat tracts. A recent study found that many long transcribed human genes were susceptible to DNA breaks leading to genetic instability at common fragile site sequences where transcription and DNA replication machinery overlap (45). RNase H1 was found to suppress this common fragile site instability supporting a role for R-loops in the instability process (45). A similar situation may arise during simultaneous transcription and DNA replication of expanded (CAG)·(CTG) repeats, which are prone to R-loop formation. While beyond the scope of the current study, the effects of concomitant DNA replication and transcription in (CAG)·(CTG) repeat instability warrant further investigation.

Expansion of the *C9orf72* (GGGGCC)·(GGCCCC) repeat has recently been demonstrated to be the leading cause of ALS/FTD. Individuals with *C9orf72* expansions show length variation between tissues of a given individual (15). The mechanism of *C9orf72* repeat expansions has yet to be studied. Interestingly, the *C9orf72* tract, is transcribed in either one or both directions (17). In all cases of repeat instability, an unusual DNA structure is believed to be involved. Here we present the novel finding that the *C9orf72* repeats can form both single- and double-R-loop structures. We also show that processing of the double-R-loop structure can lead to repeat instability, providing the first suggestion of a possible mechanism of *C9orf72* instability. The *C9orf72* tract is bidirectionally transcribed (17). We, and others recently demonstrated that the G-rich RNA strand can assume a G-quadruplex structure (28,46–47). Preliminary experiments suggest that the G-rich and C-rich strands of the *C9orf72* repeat can assume G-quadruplex and *i-motif* structures (Zamiri B, Macgregor and Pearson, unpublished data). Interestingly, herein we show that transcription across the G-rich strand yields more R-loops than across the C-rich strand (Figure 8). This may be due to R-loop stabilization by a potential G-quadruplex structure formed by the non-transcribe G-rich DNA strand (48), and/or due to the increased biophysical stability of R-loops formed with G-clusters in the transcript (2). We previously observed R-loop formation following transcription across either the (CGG)*_n_* or the (CCG)*_n_* repeats of the FRAXA locus, as well as double-R-loops for both(26). It is noteworthy that we found production of a rCGG transcript yielded more R-loops than production of the rCCG transcript, similar to our finding herein that the rGGGGCC transcript led to more R-loops than for the rGGCCCC transcript. Our results suggest that transcription may drive *C9orf72* repeat instability as it does for trinucleotide repeats (21–23).

**Figure 10.**
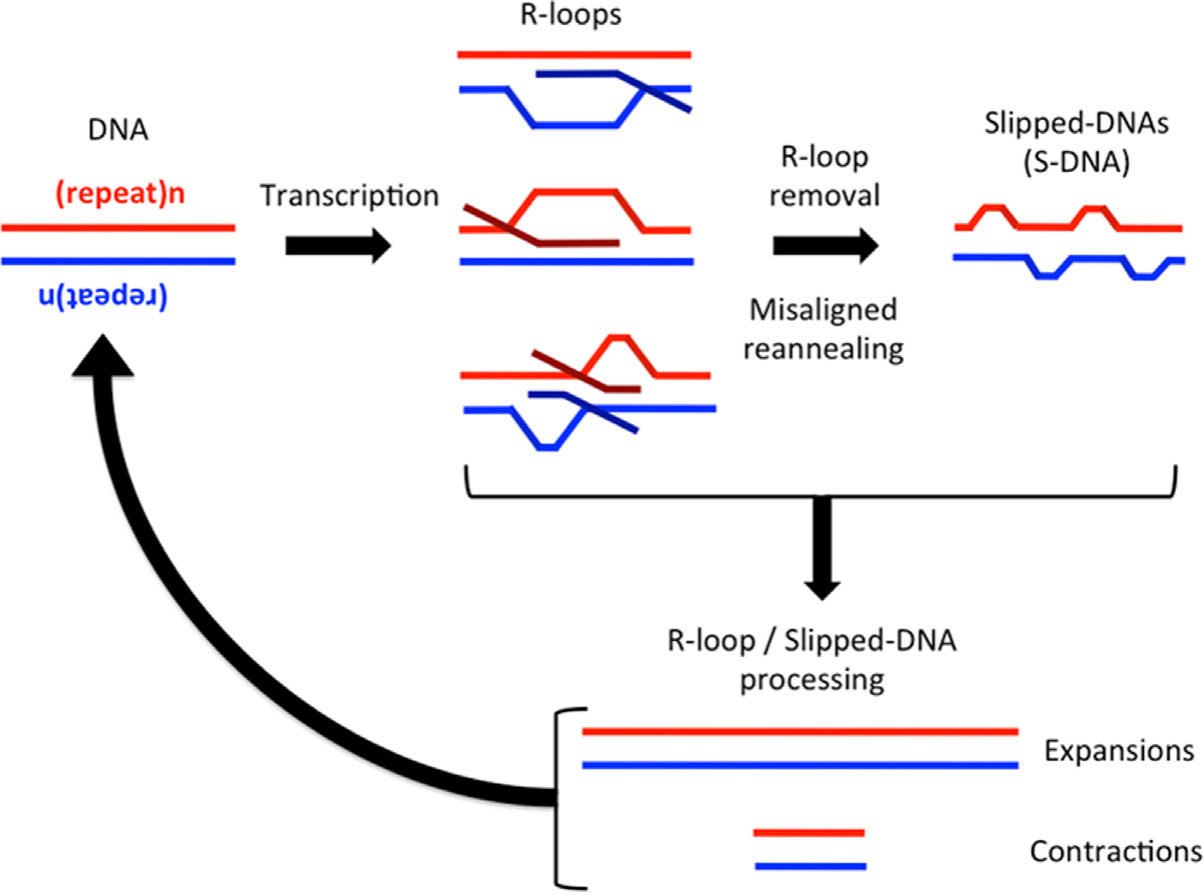
Proposed model for R-loop-mediated instability. Expanded (CAG)·(CTG) repeats are transcribed in a bidirectional manner generating rCAG-containing R-loops, rCUG-containing R-loops or double-R-loops. These products are then either directly processed by various repair proteins to generate instability or are first processed to remove the R-loop. R-loop removal may cause misaligned reannealing of the two DNA strands to generate S-DNA, which is then further processed to generate instability. This process of transcription-induced R-loop formation, R-loop processing to instability can cycle back again and again, in non-proliferating tissues, thereby continually amplifying the instability.

Repeat expansions associated with numerous neurological and neuromuscular diseases show high levels of expansions in non-proliferating tissues including specific brain regions (14,40). We found that R-loop-processing-mediated repeat instability was independent of DNA replication. Since R-loop formation occurs via transcription, which can arise in the absence of DNA replication, it is likely that instability mediated by R-loop processing can lead to instability in non-proliferating tissues and cells, such as the brain (Figure 10). That slipped S-DNAs can be an intermediate of R-loop processing via the degradation of the RNA component, suggests that human patient tissues displaying high levels of repeat instability might harbor S-DNAs at the expanded repeats. In fact, recent evidence revealed the presence of slipped-DNAs at the expanded DM1 locus in various DM1 patient tissues, where the levels of S-DNAs correlated with the levels of instability (49). Multiple cycles of transcription, R-loop formation and processing could lead to the accumulation of high levels of instability (Figure 10).

Many disease-associated repeat loci are known to be transcribed in both directions across the repeat, including the DM1, HD, SCA8, FRAXA, FRAXE and *C9orf72* loci (16). The effect of transcription direction on R-loop formation may thus be an important determinant of somatic instability based on tissue-specific differences in transcription levels and directions. Previous studies reported higher levels of instability during simultaneous convergent bidirectional transcription relative to either direction alone potentially through R-loop formation (21–23). Although the basis for R-loop-mediated instability was poorly understood, the detection of double-R-loop structures from simultaneous bidirectionally transcribed repeat templates supports a mechanism in which direct processing of these double-R-loops may generate the instability (3). We observed the highest levels of instability following processing of double-R-loops with human neuron-like cell extract relative to single-R-loops. Double-R-loop structures may be susceptible to reduced correct repair leading to instability potentially through error-prone repair as previously demonstrated for S-DNAs with repeat slip-outs (31,34). In the case of S-DNAs, there was asymmetry in repair efficiency depending on whether the slip-out sequence was CTG or CAG (where CAG slip-outs were repaired more efficiently than CTG slip-outs) (31). We and others have observed asymmetry in the formation of R-loops where rCAG R-loops form at a greater proportion than rCUG R-loops (3,11). In this study, we observe a corresponding asymmetry in R-loop-mediated instability, where rCAG R-loops trigger more instability (with a modest but consistent bias for expansions) than rCUG R-loops (with a modest but consistent bias for contractions) and rCAG+rCUG R-loops generate the greatest instability (with a significant bias for contractions). Thus, aberrant error-prone repair leading to expansions or contractions may be influenced by the structure of the slipped-out DNA formed in the R-loop, which is in turn dependent upon the direction in which the sequence is transcribed and whether it is simultaneously, bidirectionally transcribed.

It is important to determine which proteins respond to and interact with R-loops and how this generates repeat instability. One cellular factor known to be involved in trinucleotide repeat R-loop processing is RNase H (11). Lin *et al.* (11) demonstrated an increase in transcription-mediated CAG instability in human cells following shRNA knockdown of RNase H1 and H2 (the major RNase H enzymes in eukaryotes). Our results further support this finding as treatment of R-loops with RNase H prior to HeLa cell extract treatment reduced the levels of instability. Interestingly, this reduction in instability was partial relative to a non-R-loop DNA control. We found that R-loop removal with RNase H can enhance the formation of single-stranded regions in the DNA that have not properly reannealed to their complementary strand. These regions can form slipped S-DNA structures that have the potential to induce aberrant processing leading to instability, a suggestion further supported by our detection of S-DNAs following degradation of the RNA component of R-loops. Our observation is similar to findings of DNA misalignment following RNase H-mediated R-loop removal at human immunoglobulin class switch sequences (50). Subsequent aberrant processing of these S-DNAs with DNA repair complexes such as MutSβ may be what induces the partial instability observed following RNase H-mediated R-loop removal from the (CAG)·(CTG) repeat tract (34).

Transcription-mediated instability of (CAG)·(CTG) repeat tracts involves various nucleotide excision repair and mismatch repair proteins (19–20,23,51–52). How these proteins generate instability is poorly understood. An interaction between nucleotide excision repair proteins XPF-ERCC1 and XPG and R-loops was established previously for immunoglobulin class switch sequences (53). Both proteins were found to cleave R-loops formed in class switch sequences, which is proposed to be a necessary step during class switch recombination (53). We similarly demonstrated that XPF-ERCC1 and XPG proteins recognize and cleave CAG and CTG slipped structures 5’ and 3’ of the S-DNA junction, respectively (54). Subsequent error-prone DNA repair synthesis may generate instability. Binding of mismatch repair proteins and/or other repair proteins to S-DNA in the R-loop may also trigger instability through aberrant repair processes. The assessment of individual protein factors upon R-loop processing warrants further analysis.

Lin *et al.* (11) proposed a model in which R-loop formation stabilizes non-B-DNA structures in the displaced, non-template DNA strand of the R-loop, signaling various repair factors such as those of transcription-coupled repair, nucleotide excision repair and/or mismatch repair to act upon the slipped-structures thus generating aberrant repair-mediated instability. This is similar to the model proposed by Pearson *et al.* (25) in which unwinding and re-annealing of (CAG)·(CTG) repeats during DNA metabolism (including transcription) leads to the formation of slipped-, S-DNAs as a mutagenic intermediate of instability. Based on these combined findings, we propose a model in which convergent bidirectional transcription generates R-loops in various configurations that are either directly processed to trigger instability, or are first processed into S-DNA through RNA removal, which is then further processed triggering instability (Figure 10). While the level of instability we observe is modest, it represents the possible outcome of only a single round of transcription, R-loop formation and processing. Such events may occur frequently (possibly for every round of transcription) and the accumulation of such changes may amount to the high levels of instability in non-proliferating patient tissues.

In summary, we have established a novel R-loop processing assay utilizing human cell extracts. We demonstrate that double-R-loop processing generates instability of an expanded disease-associated (CAG)·(CTG) and a (GGGGCCG)·(GGCCCC) repeat tract post-transcriptionally and in the absence of DNA replication. Some of the double-R-loop-mediated instability may arise from the processing of S-DNAs that arise from the removal of RNA from R-loops.

## SUPPLEMENTARY DATA

Supplementary Data are available at NAR Online.

SUPPLEMENTARY DATA
