# Supplementary material for: Processing of double-R-loops in (CAG)·(CTG) and C9orf72 (GGGGCC)·(GGCCCC) repeats causes instability
Source: Nucleic Acids Res. 2014 Aug 21;42(16):10473–87. doi: 10.1093/nar/gku658 (PMC4176329; doi:10.1093/nar/gku658)
Supplement: SUPPLEMENTARY DATA [file supp_42_16_10473__index.html]

Processing of double-R-loops in (CAG)·(CTG) and C9orf72 (GGGGCC)·(GGCCCC) repeats causes instability — Processing of double-R-loops in (CAG)·(CTG) and C9orf72 (GGGGCC)·(GGCCCC) repeats causes instability — SUPPLEMENTARY DATA 

# Processing of double-R-loops in (CAG)·(CTG) and *C9orf72* (GGGGCC)·(GGCCCC) repeats causes instability

## SUPPLEMENTARY DATA

**Files in this Data Supplement:**

- SUPPLEMENTARY DATA
