# Supplementary material for: Processing of double-R-loops in (CAG)·(CTG) and C9orf72 (GGGGCC)·(GGCCCC) repeats causes instability
Source: Nucleic Acids Res. 2014 Aug 21;42(16):10473–87. doi: 10.1093/nar/gku658 (PMC4176329; doi:10.1093/nar/gku658)
Supplement: SUPPLEMENTARY DATA [file supp_gku658_nar-03334-f-2013-File012.pptx]

## Slide 1
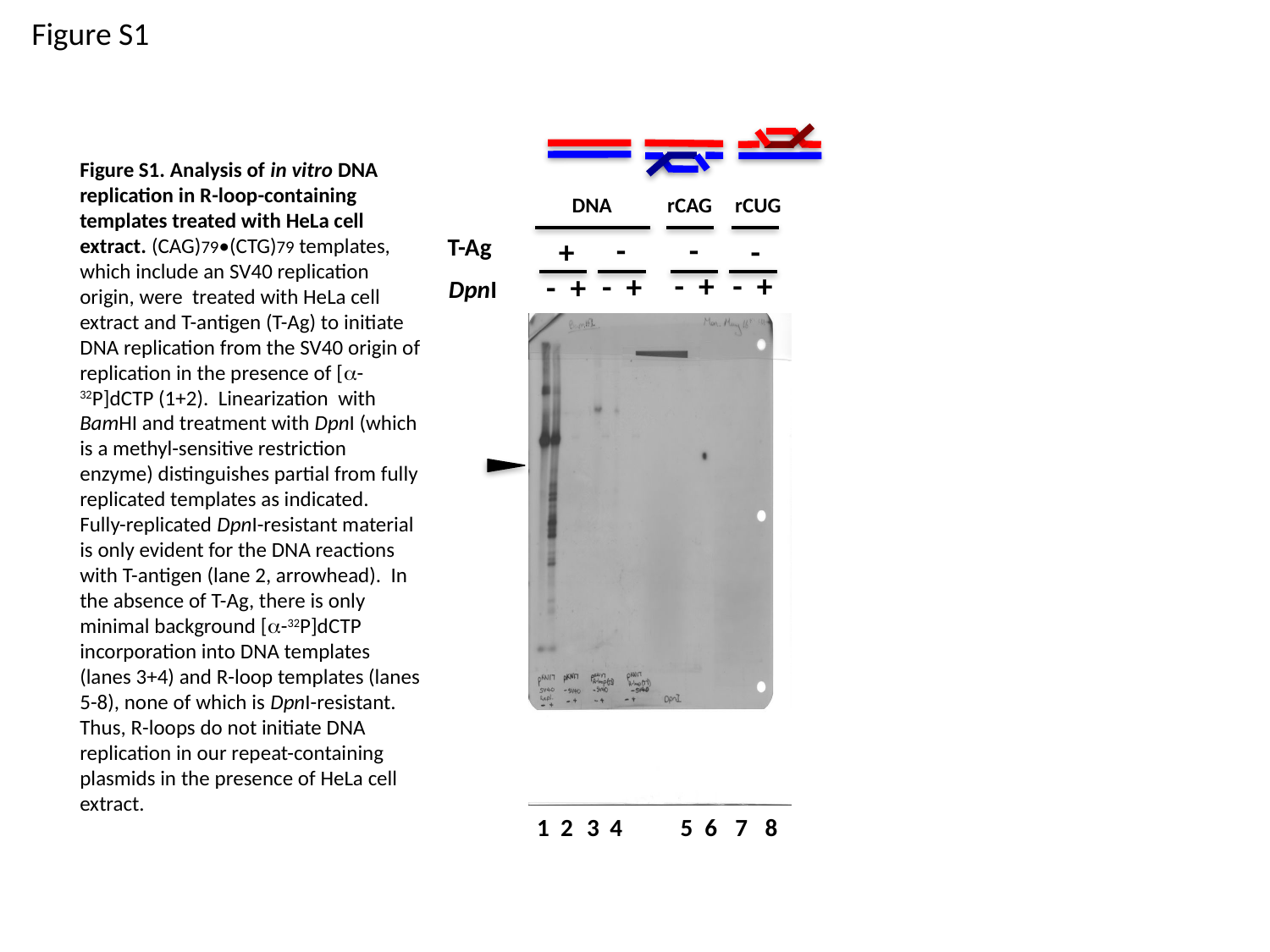

Figure S1
rCAG
rCUG
DNA
-
-
T-Ag
+
-
- +
- +
- +
- +
DpnI
1
2
3
4
5
6
7
8
Figure S1. Analysis of in vitro DNA replication in R-loop-containing templates treated with HeLa cell extract. (CAG)79•(CTG)79 templates, which include an SV40 replication origin, were treated with HeLa cell extract and T-antigen (T-Ag) to initiate DNA replication from the SV40 origin of replication in the presence of [α-32P]dCTP (1+2). Linearization with BamHI and treatment with DpnI (which is a methyl-sensitive restriction enzyme) distinguishes partial from fully replicated templates as indicated. Fully-replicated DpnI-resistant material is only evident for the DNA reactions with T-antigen (lane 2, arrowhead). In the absence of T-Ag, there is only minimal background [α-32P]dCTP incorporation into DNA templates (lanes 3+4) and R-loop templates (lanes 5-8), none of which is DpnI-resistant. Thus, R-loops do not initiate DNA replication in our repeat-containing plasmids in the presence of HeLa cell extract.

## Slide 2
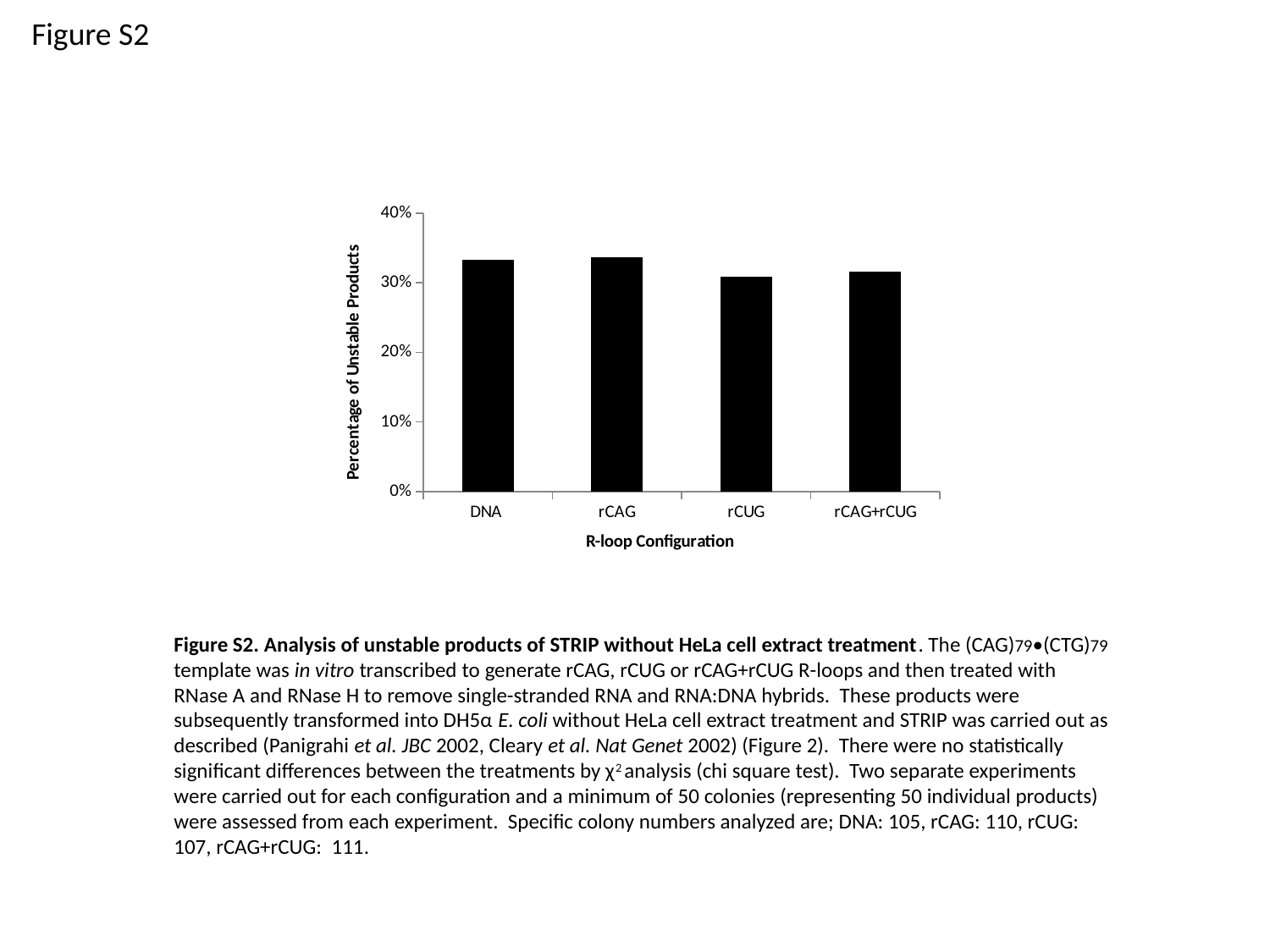

Figure S2
### Chart
| Category | |
|---|---|
| DNA | 0.3333 |
| rCAG | 0.3364 |
| rCUG | 0.3084 |
| rCAG+rCUG | 0.3153 |Figure S2. Analysis of unstable products of STRIP without HeLa cell extract treatment. The (CAG)79•(CTG)79 template was in vitro transcribed to generate rCAG, rCUG or rCAG+rCUG R-loops and then treated with RNase A and RNase H to remove single-stranded RNA and RNA:DNA hybrids. These products were subsequently transformed into DH5α E. coli without HeLa cell extract treatment and STRIP was carried out as described (Panigrahi et al. JBC 2002, Cleary et al. Nat Genet 2002) (Figure 2). There were no statistically significant differences between the treatments by χ2 analysis (chi square test). Two separate experiments were carried out for each configuration and a minimum of 50 colonies (representing 50 individual products) were assessed from each experiment. Specific colony numbers analyzed are; DNA: 105, rCAG: 110, rCUG: 107, rCAG+rCUG: 111.

## Slide 3
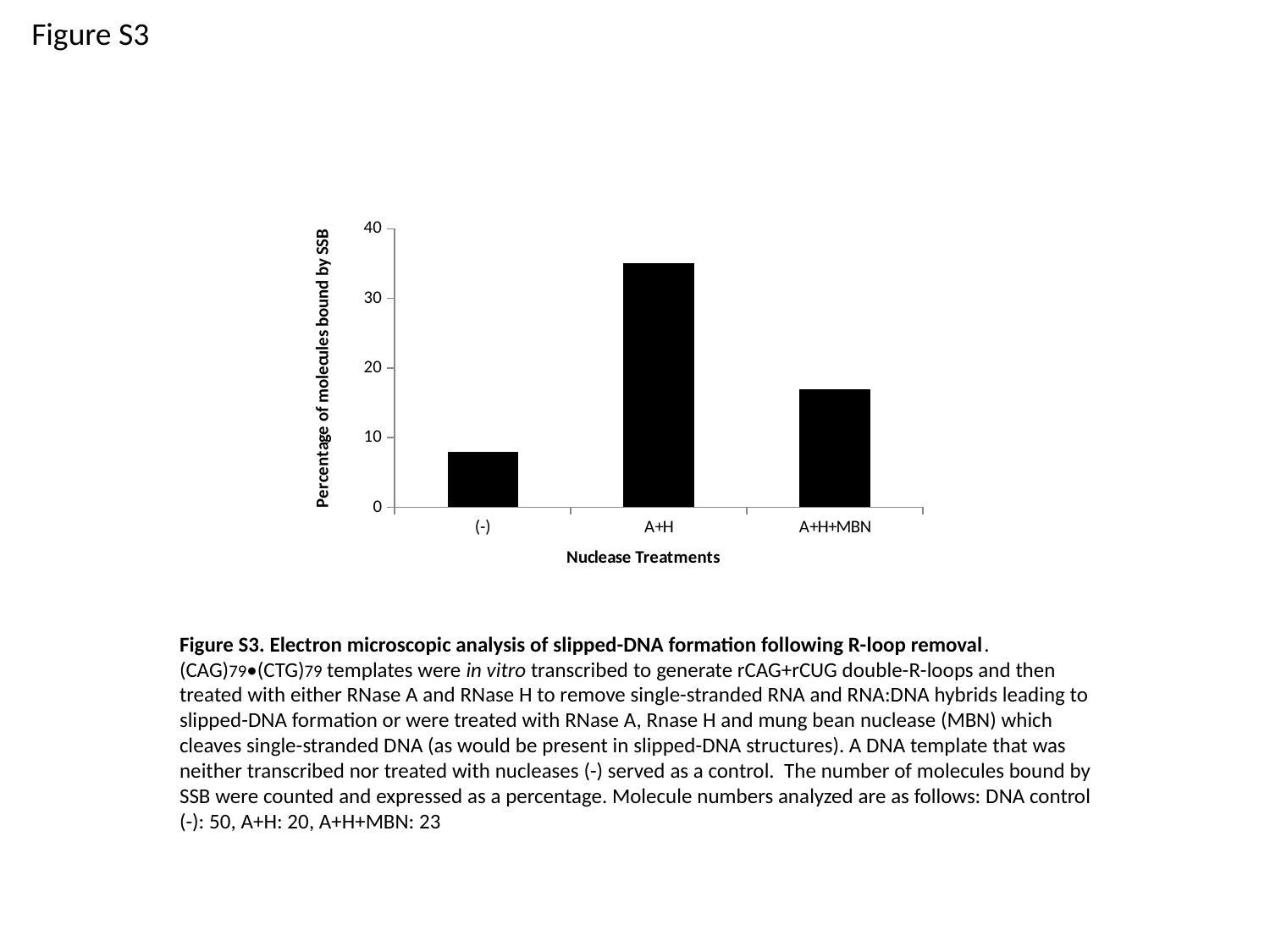

Figure S3
### Chart
| Category | |
|---|---|
| (-) | 8.0 |
| A+H | 35.0 |
| A+H+MBN | 17.0 |Figure S3. Electron microscopic analysis of slipped-DNA formation following R-loop removal. (CAG)79•(CTG)79 templates were in vitro transcribed to generate rCAG+rCUG double-R-loops and then treated with either RNase A and RNase H to remove single-stranded RNA and RNA:DNA hybrids leading to slipped-DNA formation or were treated with RNase A, Rnase H and mung bean nuclease (MBN) which cleaves single-stranded DNA (as would be present in slipped-DNA structures). A DNA template that was neither transcribed nor treated with nucleases (-) served as a control. The number of molecules bound by SSB were counted and expressed as a percentage. Molecule numbers analyzed are as follows: DNA control (-): 50, A+H: 20, A+H+MBN: 23

## Slide 4
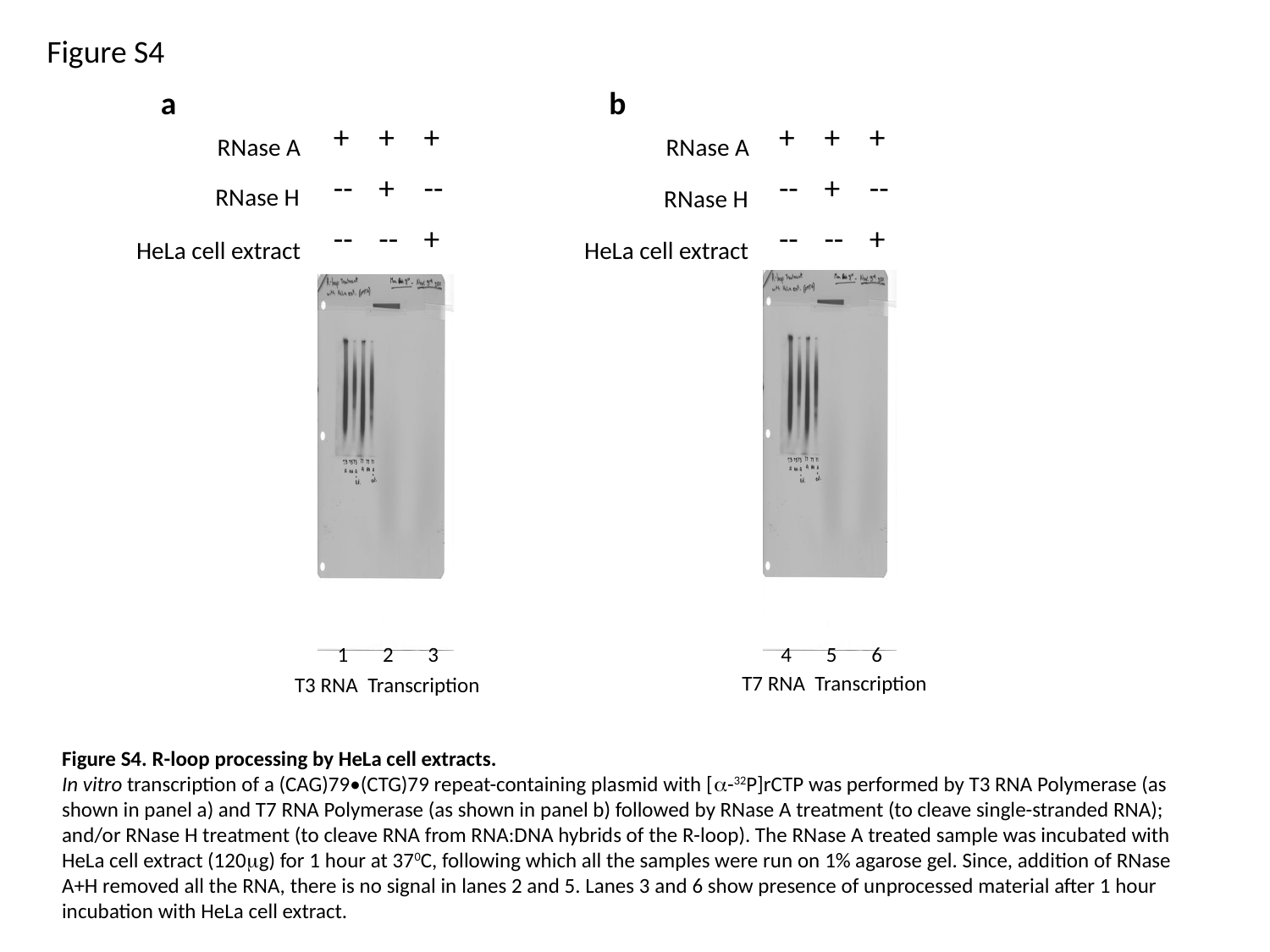

Figure S4
a
b
| + | + | + |
| --- | --- | --- |
| -- | + | -- |
| -- | -- | + |
| + | + | + |
| --- | --- | --- |
| -- | + | -- |
| -- | -- | + |
RNase A
RNase H
HeLa cell extract
RNase A
RNase H
HeLa cell extract
| 1 | 2 | 3 |
| --- | --- | --- |
| 4 | 5 | 6 |
| --- | --- | --- |
T7 RNA Transcription
T3 RNA Transcription
Figure S4. R-loop processing by HeLa cell extracts.
In vitro transcription of a (CAG)79•(CTG)79 repeat-containing plasmid with [α-32P]rCTP was performed by T3 RNA Polymerase (as shown in panel a) and T7 RNA Polymerase (as shown in panel b) followed by RNase A treatment (to cleave single-stranded RNA); and/or RNase H treatment (to cleave RNA from RNA:DNA hybrids of the R-loop). The RNase A treated sample was incubated with HeLa cell extract (120mg) for 1 hour at 370C, following which all the samples were run on 1% agarose gel. Since, addition of RNase A+H removed all the RNA, there is no signal in lanes 2 and 5. Lanes 3 and 6 show presence of unprocessed material after 1 hour incubation with HeLa cell extract.

## Slide 5
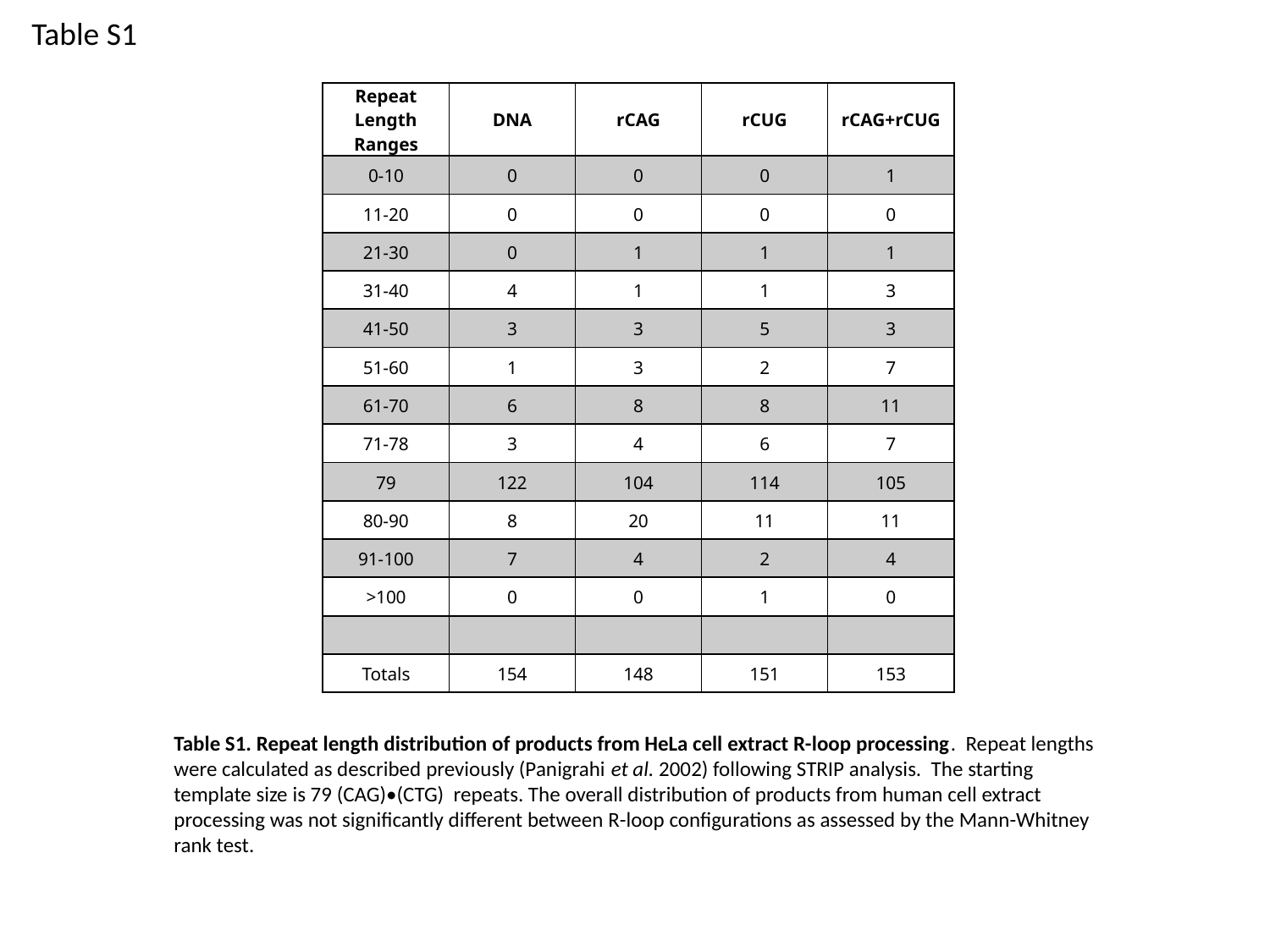

Table S1
| Repeat Length Ranges | DNA | rCAG | rCUG | rCAG+rCUG |
| --- | --- | --- | --- | --- |
| 0-10 | 0 | 0 | 0 | 1 |
| 11-20 | 0 | 0 | 0 | 0 |
| 21-30 | 0 | 1 | 1 | 1 |
| 31-40 | 4 | 1 | 1 | 3 |
| 41-50 | 3 | 3 | 5 | 3 |
| 51-60 | 1 | 3 | 2 | 7 |
| 61-70 | 6 | 8 | 8 | 11 |
| 71-78 | 3 | 4 | 6 | 7 |
| 79 | 122 | 104 | 114 | 105 |
| 80-90 | 8 | 20 | 11 | 11 |
| 91-100 | 7 | 4 | 2 | 4 |
| >100 | 0 | 0 | 1 | 0 |
| | | | | |
| Totals | 154 | 148 | 151 | 153 |
Table S1. Repeat length distribution of products from HeLa cell extract R-loop processing. Repeat lengths were calculated as described previously (Panigrahi et al. 2002) following STRIP analysis. The starting template size is 79 (CAG)•(CTG) repeats. The overall distribution of products from human cell extract processing was not significantly different between R-loop configurations as assessed by the Mann-Whitney rank test.

## Slide 6
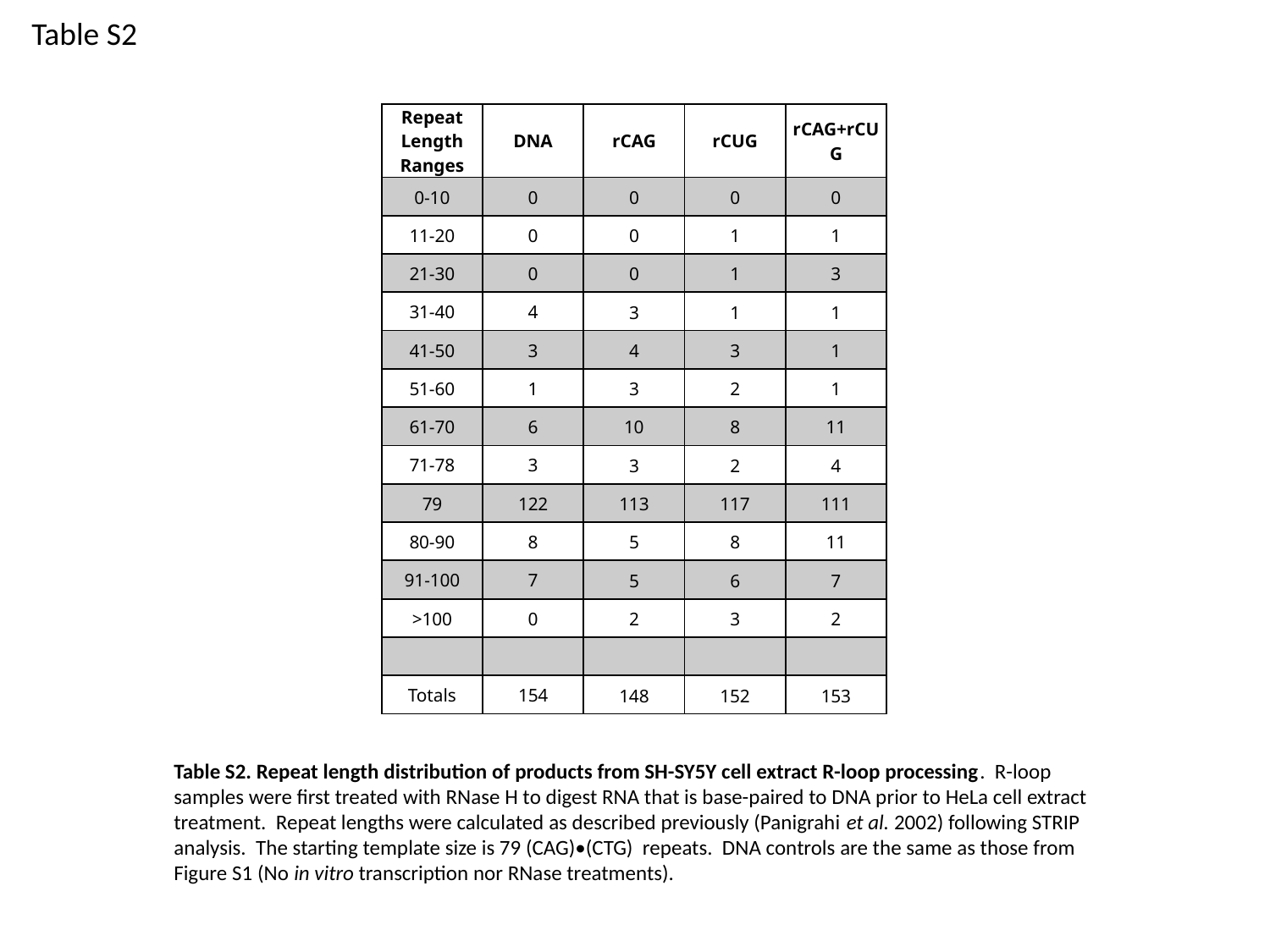

Table S2
| Repeat Length Ranges | DNA | rCAG | rCUG | rCAG+rCUG |
| --- | --- | --- | --- | --- |
| 0-10 | 0 | 0 | 0 | 0 |
| 11-20 | 0 | 0 | 1 | 1 |
| 21-30 | 0 | 0 | 1 | 3 |
| 31-40 | 4 | 3 | 1 | 1 |
| 41-50 | 3 | 4 | 3 | 1 |
| 51-60 | 1 | 3 | 2 | 1 |
| 61-70 | 6 | 10 | 8 | 11 |
| 71-78 | 3 | 3 | 2 | 4 |
| 79 | 122 | 113 | 117 | 111 |
| 80-90 | 8 | 5 | 8 | 11 |
| 91-100 | 7 | 5 | 6 | 7 |
| >100 | 0 | 2 | 3 | 2 |
| | | | | |
| Totals | 154 | 148 | 152 | 153 |
Table S2. Repeat length distribution of products from SH-SY5Y cell extract R-loop processing. R-loop samples were first treated with RNase H to digest RNA that is base-paired to DNA prior to HeLa cell extract treatment. Repeat lengths were calculated as described previously (Panigrahi et al. 2002) following STRIP analysis. The starting template size is 79 (CAG)•(CTG) repeats. DNA controls are the same as those from Figure S1 (No in vitro transcription nor RNase treatments).

## Slide 7
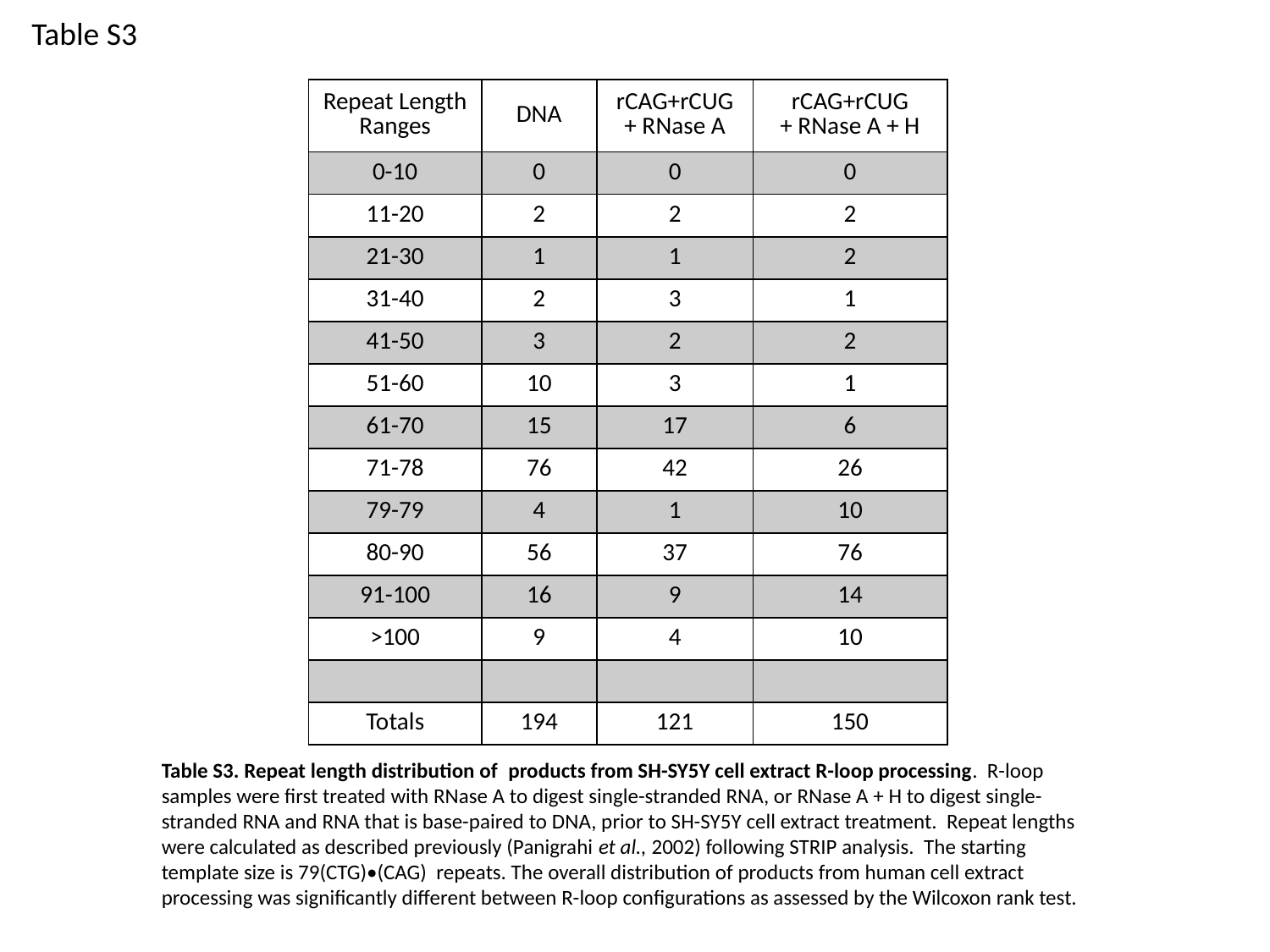

Table S3
| Repeat Length Ranges | DNA | rCAG+rCUG + RNase A | rCAG+rCUG + RNase A + H |
| --- | --- | --- | --- |
| 0-10 | 0 | 0 | 0 |
| 11-20 | 2 | 2 | 2 |
| 21-30 | 1 | 1 | 2 |
| 31-40 | 2 | 3 | 1 |
| 41-50 | 3 | 2 | 2 |
| 51-60 | 10 | 3 | 1 |
| 61-70 | 15 | 17 | 6 |
| 71-78 | 76 | 42 | 26 |
| 79-79 | 4 | 1 | 10 |
| 80-90 | 56 | 37 | 76 |
| 91-100 | 16 | 9 | 14 |
| >100 | 9 | 4 | 10 |
| | | | |
| Totals | 194 | 121 | 150 |
Table S3. Repeat length distribution of products from SH-SY5Y cell extract R-loop processing. R-loop samples were first treated with RNase A to digest single-stranded RNA, or RNase A + H to digest single-stranded RNA and RNA that is base-paired to DNA, prior to SH-SY5Y cell extract treatment. Repeat lengths were calculated as described previously (Panigrahi et al., 2002) following STRIP analysis. The starting template size is 79(CTG)•(CAG) repeats. The overall distribution of products from human cell extract processing was significantly different between R-loop configurations as assessed by the Wilcoxon rank test.

## Slide 8
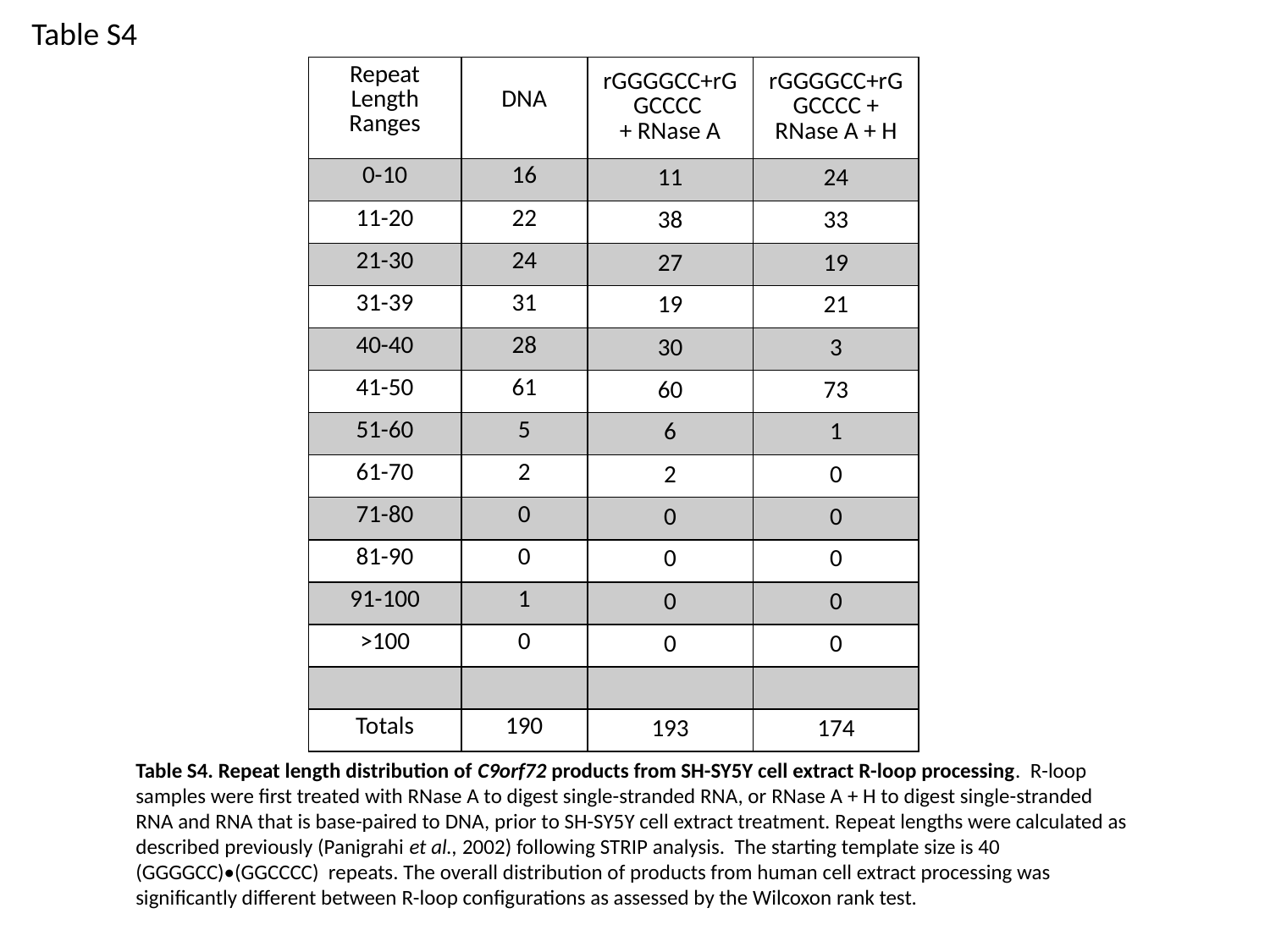

Table S4
| Repeat Length Ranges | DNA | rGGGGCC+rGGCCCC + RNase A | rGGGGCC+rGGCCCC + RNase A + H |
| --- | --- | --- | --- |
| 0-10 | 16 | 11 | 24 |
| 11-20 | 22 | 38 | 33 |
| 21-30 | 24 | 27 | 19 |
| 31-39 | 31 | 19 | 21 |
| 40-40 | 28 | 30 | 3 |
| 41-50 | 61 | 60 | 73 |
| 51-60 | 5 | 6 | 1 |
| 61-70 | 2 | 2 | 0 |
| 71-80 | 0 | 0 | 0 |
| 81-90 | 0 | 0 | 0 |
| 91-100 | 1 | 0 | 0 |
| >100 | 0 | 0 | 0 |
| | | | |
| Totals | 190 | 193 | 174 |
Table S4. Repeat length distribution of C9orf72 products from SH-SY5Y cell extract R-loop processing. R-loop samples were first treated with RNase A to digest single-stranded RNA, or RNase A + H to digest single-stranded RNA and RNA that is base-paired to DNA, prior to SH-SY5Y cell extract treatment. Repeat lengths were calculated as described previously (Panigrahi et al., 2002) following STRIP analysis. The starting template size is 40 (GGGGCC)•(GGCCCC) repeats. The overall distribution of products from human cell extract processing was significantly different between R-loop configurations as assessed by the Wilcoxon rank test.
